# Probing DFT Functionals in the Analysis of Enthalpy and Gibbs Free Energy: A Case Study of a Heptakis(2,6-di-O-methyl)-β-cyclodextrin Complex with a Novel Fluorinated Compound

**DOI:** 10.3390/molecules31091420

**Published:** 2026-04-25

**Authors:** Marta Hoelm, Zdzisław Kinart

**Affiliations:** Department of Physical Chemistry, Faculty of Chemistry, University of Lodz, Pomorska 163/165, 90-236 Lodz, Poland

**Keywords:** cyclodextrins, drug delivery, enthalpy, Gibbs free energy, DFT calculations, r^2^SCAN-3c, solvent models, conductometry

## Abstract

In this study, we evaluated various density functional theory (DFT) methods to obtain thermodynamic parameters, such as enthalpy and Gibbs free energy, and compared them with experimental values obtained from conductometric analysis. As a model system, we chose the heptakis(2,6-di-O-methyl)-β-cyclodextrin (DIMEB) complex with the recently synthesized fluorinated compound, butane-1,4-diyl bis(2,2,2-trifluoroethane-1-sulfonate) (BFS). The analysis was carried out in the temperature range of 293.15–313.15 K. A conformational search was performed to identify the most stable complexes. The final stage of optimization was conducted at the ωB97X-D4/6-31G(d,p) level of theory in the presence of water, modeled using the conductor-like polarizable continuum model (CPCM). The thermodynamic analysis indicates that almost all theoretical methods overestimate the enthalpy and Gibbs free energy. This also applies to Minnesota functionals, which are commonly recommended for thermochemistry studies. The best agreement with experimental results was obtained for the composite methods r^2^SCAN-3c and PBEh-3c, with the coefficient of determination (R^2^ = 0.9972) indicating excellent correlation between r^2^SCAN-3c and experiment.

## 1. Introduction

Drug delivery systems (DDS) represent an important and rapidly evolving area of pharmaceutical research, aimed at improving the efficiency and safety of therapeutic agents. These systems are designed to transport drugs to specific sites in the body in a controlled and predictable manner, enabling modulation of drug release profiles, bioavailability, and biological interactions [1]. In recent years, significant attention has been devoted to the development of advanced DDS, particularly those based on nanotechnology. Nanocarriers have emerged as one of the most extensively studied platforms due to their tunable physicochemical properties and ability to improve drug stability and targeting [1]. Various nanostructures, including nanocages, nanosheets, and nanotubes, have been investigated using both experimental and theoretical approaches. For example, hybrid nanocages based on boron, carbon, and nitrogen exhibit enhanced adsorption capacity and favorable thermodynamic properties for drug binding, indicating their potential as efficient carriers [2]. Similarly, graphene oxide nanosheets have been shown to interact strongly with bioactive molecules such as curcumin through non-covalent interactions [3].

Other nanostructured systems, such as MgO nanotubes and silicon-based nanotubes, have also demonstrated promising drug encapsulation and release properties. The computational studies reveal that drug loading in such carriers is typically thermodynamically favorable and governed by non-covalent interactions, with Gibbs free energy values confirming spontaneous complex formation [4,5]. Furthermore, novel materials like graphdiyne (2D carbon allotrope) have been proposed as next-generation nanocarriers, offering enhanced reactivity, tunable electronic properties, and strong charge-transfer interactions [6].

Beyond nanocarriers, alternative DDS platforms are also being intensively explored, including microneedle-based systems, which enable minimally invasive and targeted drug administration. These systems can improve drug penetration and provide sustained release, particularly in challenging applications such as ocular therapy [7]. In addition, a wide range of supramolecular and polymer-based carriers, such as fullerenes [8], cubosomes [9], calixarenes [10], cellulose-derived systems [11], and cryptands [12,13], continue to expand the diversity of DDS under investigation.

Among supramolecular carriers, cyclodextrins (CDs) occupy a prominent position due to their ability to form inclusion complexes with a variety of guest molecules. Their hydrophobic cavity enables encapsulation of poorly soluble drugs, improving their physicochemical and biological properties without chemical modification. For instance, β-cyclodextrin complexes with anticancer agents have been shown to be thermodynamically stable, as evidenced by favorable Gibbs free energy values [14].

Particular attention has been given to β-cyclodextrin derivatives, such as heptakis(2,6-di-O-methyl)-β-cyclodextrin (DIMEB), which often exhibit improved solubility and binding characteristics compared to native CDs. Studies indicate that DIMEB forms stable inclusion complexes with various pharmaceutical compounds, with binding driven by a combination of hydrophobic interactions and hydrogen bonding [15,16]. Additionally, DIMEB-based systems can significantly enhance drug solubility, stability, and bioavailability, while maintaining low cytotoxicity, confirming their suitability as safe drug carriers [17,18].

Despite the extensive research on cyclodextrin-based DDS, there is still a lack of systematic studies evaluating the performance of different density functional theory (DFT) methods in describing their thermochemical properties.

**Figure 1:**
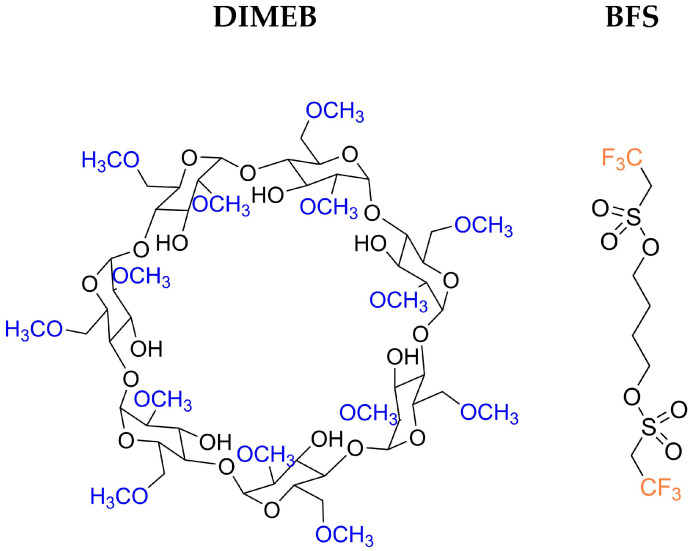
The schematic representation of Heptakis(2,6-di-O-methyl)-β-cyclodextrin (DIMEB) and butane-1,4-diyl bis(2,2,2-trifluoroethane-1-sulfonate) (BFS).

Therefore, in this work, we present a combined theoretical and experimental investigation of the inclusion complex formed between DIMEB and a recently synthesized compound, butane-1,4-diyl bis(2,2,2-trifluoroethane-1-sulfonate) (BFS) [19] (Figure 1). The primary objective is to evaluate the performance of various DFT functionals in describing key thermodynamic parameters, including enthalpy and Gibbs free energy, for the DIMEB:BFS complex. Experimental data obtained from conductometric measurements in the temperature range of 293.15–313.15 K are compared with theoretical predictions. The results indicate that the complex formation is thermodynamically favorable and spontaneous, with an exothermic character, which is consistently reproduced by the applied computational methods.

## 2. Results and Discussion

### 2.1. Analysis of Structural Properties of DIMEB:BFS Complex—Theoretical Investigation

**Figure 2:**
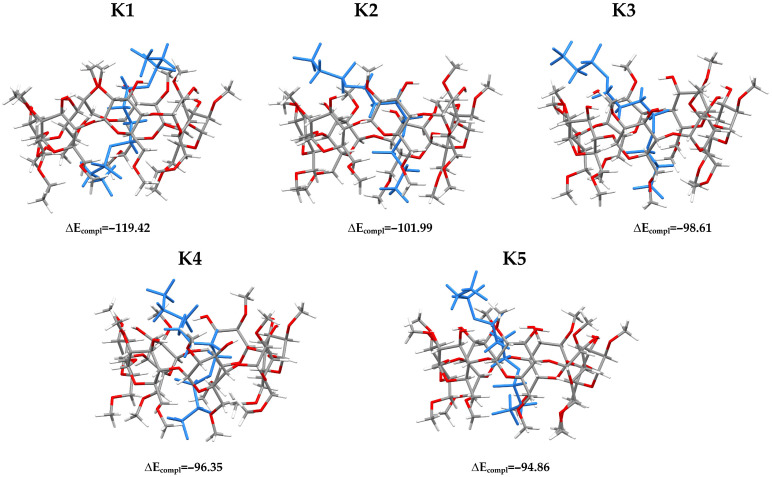
The most stable complexes of DIMEB:BFS obtained at the ωB97X-D4/6-31G(d,p) theory level using the CPCM solvent model (water). BFS is highlighted in blue. ΔE_compl_ indicates complexation energy [kJ/mol], corrected for basis set superposition error (BSSE) using the counterpoise method. The total energy values and coordinates are listed in Tables S2 and S3, respectively. Atom color scheme: carbon—dark gray, oxygen—red, hydrogen—light gray.

The searching of the most stable complex of DIMEB:BFS at the last stage of the configurational analysis was performed at the ωB97X-D4/6-31G(d,p) theory level. The most stable complexes are presented in Figure 2. In each configuration, DIMEB was oriented such that two oxygen atoms (on the left side, i.e., the wider rim), O20 and O182, belonging to the hydroxymethyl groups attached to the C2 carbon atoms, were superimposed. Although three initial configurations (O1, O2, and O3; see Figure S1) were considered during the construction of the starting complex models, after all stages of configurational analysis, the complexes ultimately converged to a single configuration, namely O1. This results from the interactions that occurred during the complex formation. In general, BFS forms interactions with DIMEB by creating C-H···O hydrogen bonds (HB). In almost all complexes 8 HB is observed, with the exception of K2 and K5, which have 9 and 6 respectively (see Table S1).

**Figure 3:**
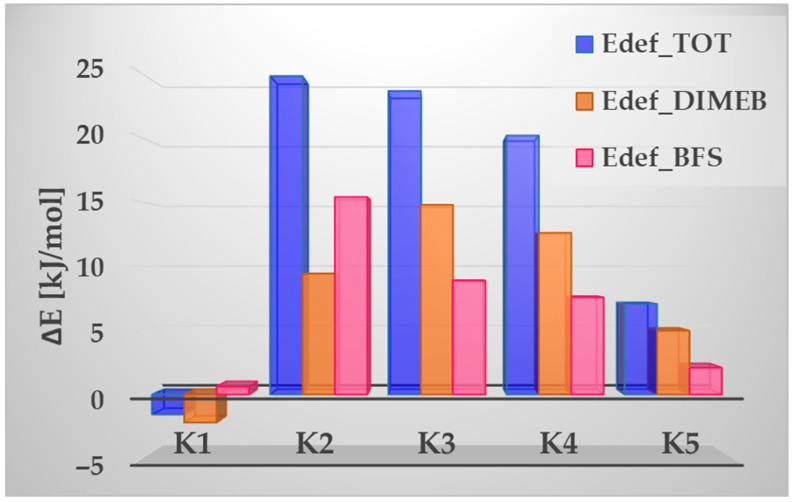
Deformation energies calculated for BFS (E_def_BFS_), DIMEB (E_def_DIMEB_), and the total deformation energy (E_def_TOT_).

The number of hydrogen bonds can be related to the deformation energies, the values of which are presented in Figure 3 for BFS (E_def_BFS_), DIMEB (E_def_DIMEB_), and the total deformation energy (E_def_TOT_). The deformation energy is defined as the energy required to distort a molecule from its optimal geometry to the geometry it adopts in the complex. Higher deformation energies indicate larger structural changes relative to the isolated molecules. The highest deformation energy of BFS is observed for the K2 complex, in which BFS forms the largest number of hydrogen bonds with DIMEB. In contrast, the lowest values are observed for K5 (where the smallest number of HBs between BFS and DIMEB is found) and for K1. In the latter case, a specific situation is observed: the deformation energy for DIMEB, and consequently the total deformation energy, have negative values. This indicates that the geometry of DIMEB is more stable than its corresponding isolated structure.

For the K5 complex, a relatively high interaction energy (ΔE_int_) is observed, amounting to –101.92 kJ/mol, while the strongest interaction occurs for K2, with a value of –126.78 kJ/mol. In turn, for the most stable complex, K1, ΔE_int_ equals –117.87 kJ/mol and is similar to the value of –116.50 kJ/mol observed for K4. The correlations between the number of hydrogen bonds and the deformation, interaction, and complexation energies are summarized in Table S1.

**Figure 4:**
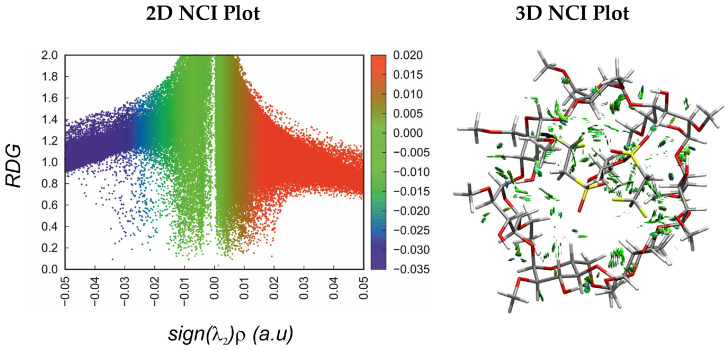
2D NCI scatter plots (**left**) and 3D reduced density gradient isosurfaces (**right**) for the most stable complex K1.

To characterize the nature of the interactions, we performed a non-covalent interaction (NCI) analysis. The NCI isosurface is presented only for the K1 complex as a representative model (see Figure 4), since a similar trend is observed for the other complexes. In general, as expected, the 3D NCI plot is dominated by green surfaces, which correspond to weak interaction regions associated with van der Waals dispersion forces. Strong attractive interactions, represented by the blue color, correspond to intramolecular hydrogen bonding (between hydroxyl groups of DIMEB).

Figure 2 also presents the complexation energies (ΔE_compl_), which serve as a measure of complex stability: the more negative the value, the more stable the complex. All values are corrected for basis set superposition error (BSSE) using the counterpoise method. As expected, DIMEB forms very stable inclusion complexes. These values can be compared with the literature data. BFS was recently investigated in our previous work [13], in which another molecule, 1,10-*N,N′*-bis-(β-D-ureidoglucosyl)-4,7,13,16-tetraoxa-1,10-diazacyclopentadecane (SMT), was studied as a potential drug carrier for BFS. Among the various computational methods applied, the ωB97X-D4/6-31++G(d,p) level of theory with the CPCM solvent model was used. In the present study, we employed a smaller basis set, 6-31G(d,p), because DIMEB contains a significantly larger number of atoms than SMT. Nevertheless, we recalculated the energy of the SMT:BFS complex at the ωB97X-D4/6-31G(d,p) level to obtain the complexation energy at the same level of theory as used in this work. The obtained ΔE_compl_ value was –39.52 kJ/mol, which is significantly less negative than in the case of the DIMEB:BFS complexes. However, the strategy of configurational search used in our previous work differed substantially from that applied here. In ref. [13], only the most stable conformers of BFS and SMT obtained from conformational analysis were used to construct the initial models of the complexes. In its most stable geometry, BFS adopts a bent conformation, whereas in the present work we used experimental coordinates obtained from the Cambridge Structural Database (CSD; see Section 3). In the solid state, BFS adopts a linear geometry, as shown in Figure S1, where the initial orientations of the complexes are presented. Moreover, SMT is capable of forming only non-inclusion complexes, as it does not possess a well-defined cavity like DIMEB. In ref. [20], it was clearly demonstrated that cyclodextrins (CDs) form significantly more stable inclusion complexes than non-inclusion ones.

### 2.2. The Influence of DFT Functionals on the Thermochemistry Parameters-Comparison with the Experimental Study

For five conformers identified at the ωB97X-D4/6-31G(d,p) level (Figure 2), a series of DFT functionals was tested to obtain the enthalpy (ΔH) and Gibbs free energy (ΔG) of the complexation process. The following methods were selected: M06-2X-D3/6-31G(d,p) (PCM), M05-2X-D3/6-31G(d,p) (PCM), M08-HX-D3/6-31G(d,p) (PCM), MN15-L-D3/6-31G(d,p) (PCM), BHandHLYP-D4/6-31G(d,p) (CPCM), CAM-B3LYP-D4/6-31G(d,p) (CPCM), r^2^SCAN50-D4/6-31G(d,p) (CPCM), ωB97X-D4/6-31G(d,p) (CPCM), B3LYP-D4/6-31G(d,p) (CPCM), r^2^SCAN-3c (CPCM), and PBEh-3c (CPCM). The r^2^SCAN-3c and PBEh-3c methods are composite approaches, in which the functional is combined with a predefined basis set. For r^2^SCAN-3c, the def2-mTZVPP basis set is used, whereas PBEh-3c employs the def2-mSVP basis set.

**Figure 5:**
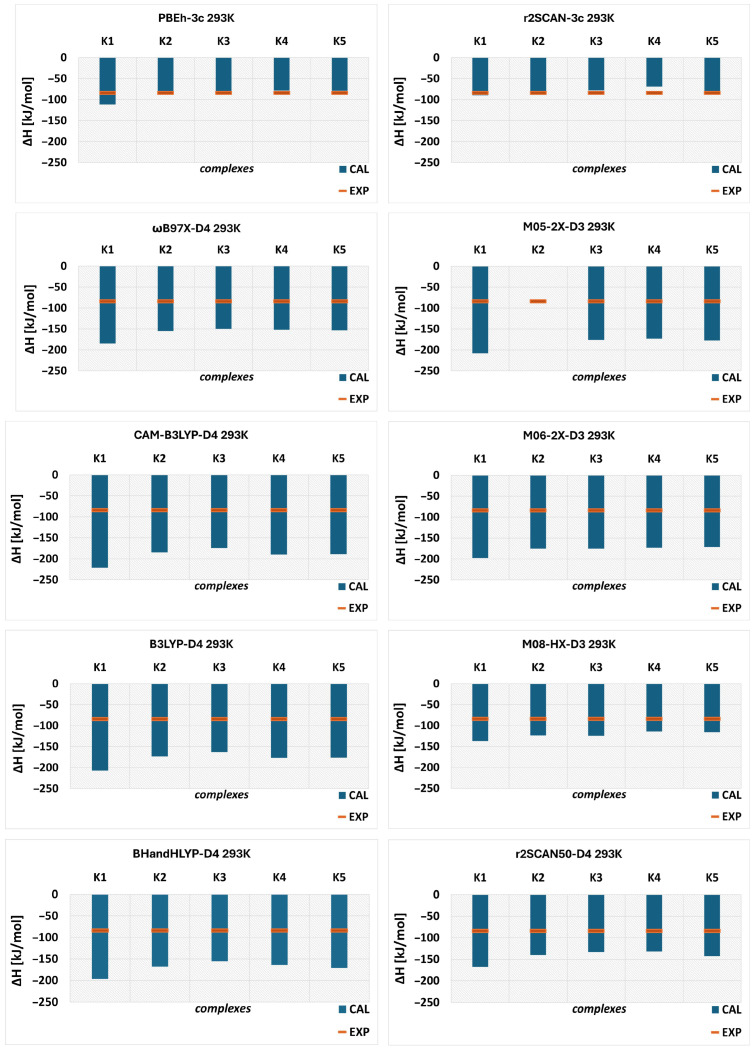
Enthalpy (ΔH) of the complexation process for the DIMEB:BFS complex obtained from theoretical calculations (CAL) and experimental measurements (EXP) at 293.15 K.

**Figure 6:**
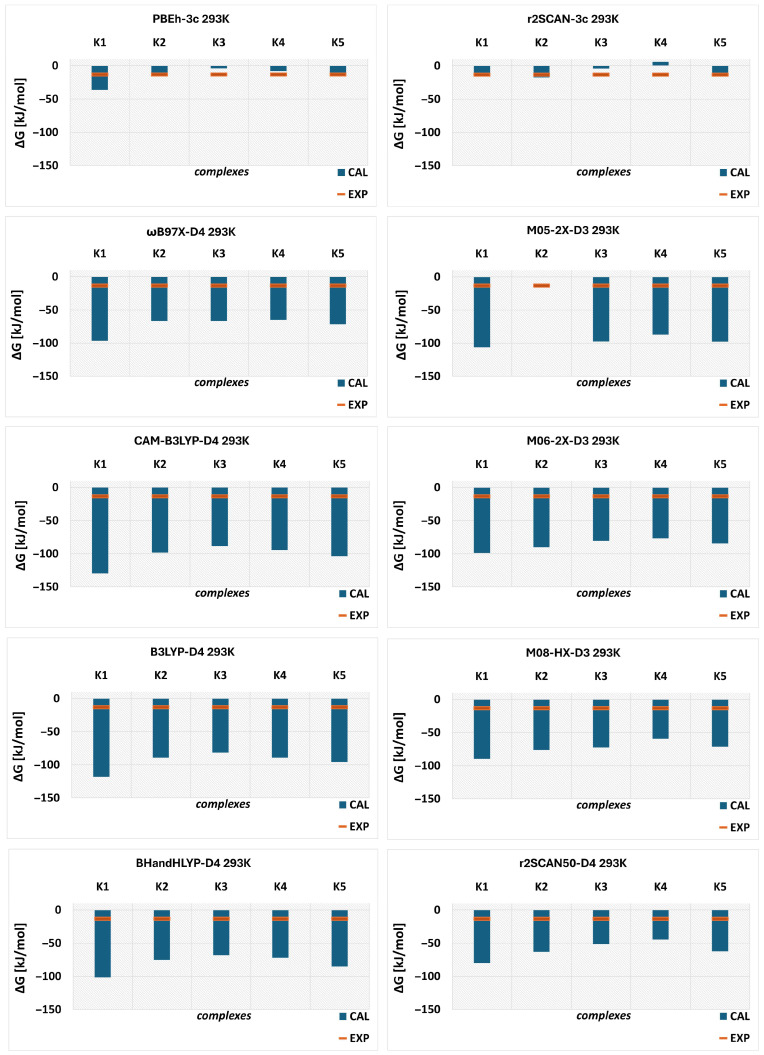
Gibbs free energy (ΔG) of the complexation process for the DIMEB:BFS complex obtained from theoretical calculations (CAL) and experimental measurements (EXP) at 293.15 K.

Initially, each conformer optimized at the ωB97X-D4/6-31G(d,p) level was reoptimized at the respective level of theory (listed above), followed by vibrational frequency calculations. These calculations were performed in the temperature range of 293.15–313.15 K. Experimental values were obtained from conductometric analysis. The results for ΔH and ΔG at 293.15 K are presented in Figure 5 and Figure 6, respectively, while the data for other temperatures are summarized in Tables S4–S14.

In general, the experimental results (EXP) indicate that the complexation process is spontaneous and exothermic, as evidenced by the negative values of ΔG and ΔH, respectively (see Figure 5 and Figure 6). This trend is maintained across the entire temperature range (293.15–313.15 K), although a more detailed discussion is provided below. All computational methods are consistent with the experimental findings, with a minor exception observed for K4 calculated using the r^2^SCAN-3c method, for which the Gibbs free energy is positive (Figure 6). However, the computational results significantly overestimate the thermodynamic parameters. The ωB97X-D4/6-31G(d,p) method, used to determine the energetic minima, overestimates these parameters for all configurations. Minnesota functionals are often recommended for thermochemical calculations; however, in the present case, they also yield overestimated values. For instance, among the tested methods, M08-HX provides the smallest deviations for enthalpy, although they remain substantial.

In the case of M05-2X, no values were obtained for K2 due to the inability to optimize the geometry of this conformer at this level of theory, despite multiple attempts. A similar issue was encountered for MN15-L, for which results could not be obtained for two complexes (K2 and K4; see Table S13), and therefore this method is not included in the Figure 5 and Figure 6. Nevertheless, for the most stable complex, MN15-L yields ΔH = –212.68 kJ/mol, which differs significantly from the experimental value (–84.10 kJ/mol). A similar trend is observed for Gibbs free energy.

In contrast, the r^2^SCAN-3c and PBEh-3c methods provide values much closer to the experimental data, particularly for Gibbs free energy (Figure 6). The best agreement is observed for the K5 complex, which is the least stable structure according to ωB97X-D4. Notably, K5 also shows the smallest deviation from experimental enthalpy values. For Gibbs free energy, PBEh-3c likewise identifies K5 as the system with the best agreement. In the case of enthalpy, PBEh-3c indicates comparable agreement for three complexes: K5, K2, and K1.

**Figure 7:**
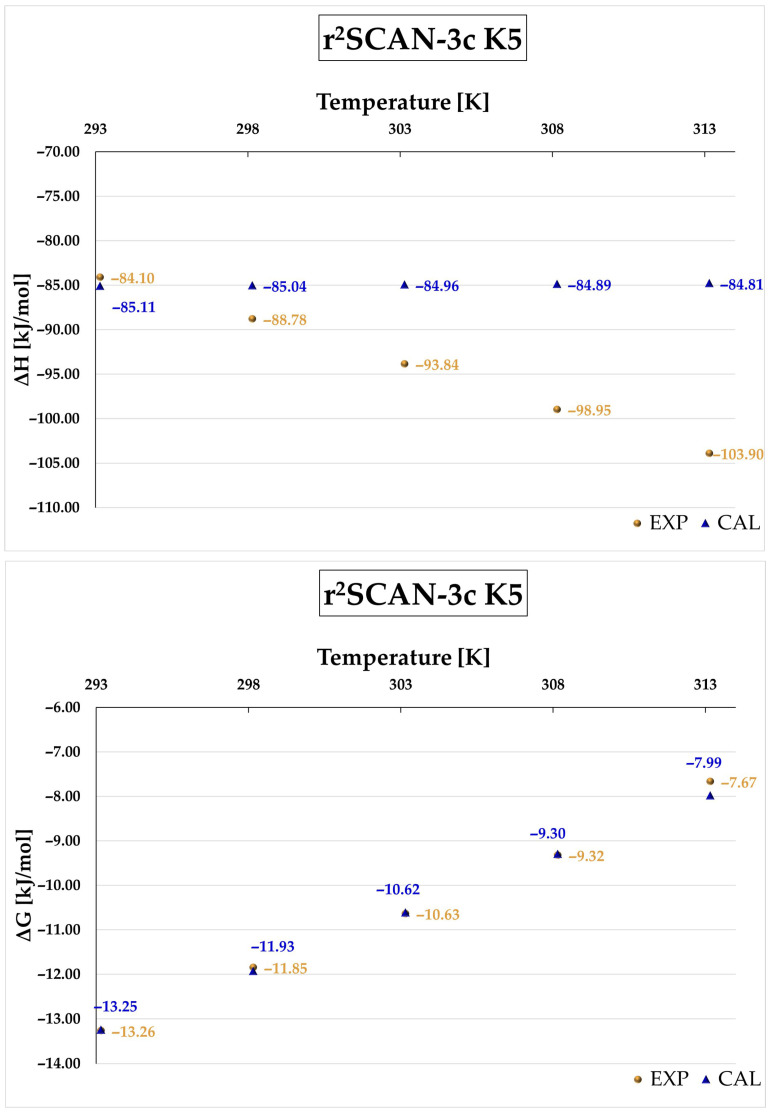
Enthalpy (ΔH) and Gibbs free energy (ΔG) of the complexation process for the DIMEB:BFS complex, obtained from theoretical calculations (CAL) at the r^2^SCAN-3c level for the K5 complex and from experimental measurements (EXP) over the temperature range 293.15–313.15 K.

**Figure 8:**
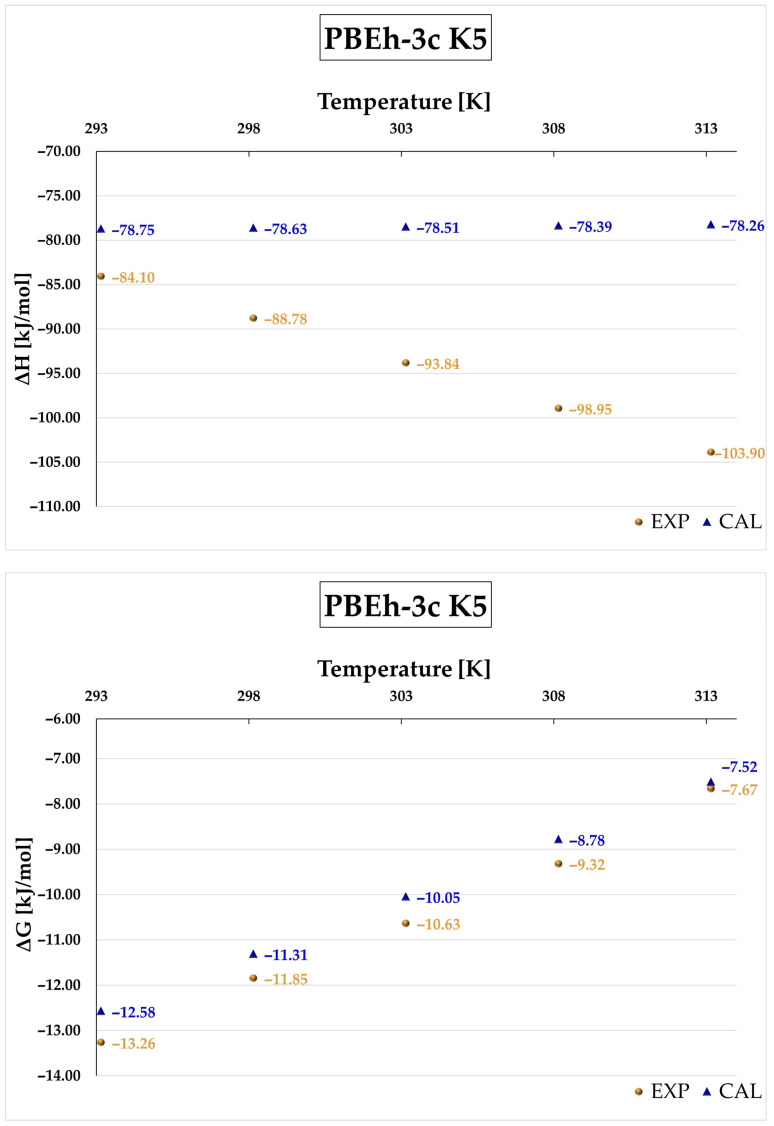
Enthalpy (ΔH) and Gibbs free energy (ΔG) of the complexation process for the DIMEB:BFS complex, obtained from theoretical calculations (CAL) at the PBEh-3c level for the K5 complex and from experimental measurements (EXP) over the temperature range 293.15–313.15 K.

Figure 7 presents the temperature dependence of ΔH and ΔG for the K5 complex calculated at the r^2^SCAN-3c level in the range 293–313 K. A very good agreement with experimental data is observed for Gibbs free energy, as confirmed by the coefficient of determination (R^2^ = 0.9972). In contrast, the calculated enthalpy remains nearly constant with temperature, which is inconsistent with the experimental trend. A similar behavior is observed for the PBEh-3c method (see Figure 8 and Figure S2). While good agreement is also obtained for Gibbs free energy, r^2^SCAN-3c provides slightly better accuracy due to smaller deviations.

Finally, it should be emphasized that although the DIMEB:BFS complex investigated in this study is of moderate size, it represents a highly flexible system. Moreover, only implicit solvent models were considered, which do not account for specific interactions such as hydrogen bonding between solvent and solute. In the case of cyclodextrins, such interactions are known to play an important role [21]. Nevertheless, among the eleven tested theoretical methods, only r^2^SCAN-3c and PBEh-3c provide results in close agreement with the experimental data. The remaining methods systematically overestimate thermodynamic parameters such as enthalpy and Gibbs free energy. The superior performance of the r^2^SCAN-3c and PBEh-3c composite methods observed in this work can be attributed to their balanced and system-specific design. Unlike conventional DFT calculations employed in this work, which use a fixed 6-31G(d,p) basis set, these composite methods are constructed as integrated frameworks combining a density functional, a specially tailored medium-sized basis set, and additional correction schemes. For instance, r^2^SCAN-3c includes the D4 dispersion correction and the geometrical counterpoise (gCP) correction, which significantly improve the description of noncovalent interactions by accounting for dispersion effects and residual basis set superposition error. Moreover, the modified mTZVPP basis employed in r^2^SCAN-3c includes enhanced polarization functions on hydrogen and heteroatoms (N, F), which is crucial for accurately describing hydrogen-bonding interactions that dominate host–guest complexes.

Comparing both composite methods (r^2^SCAN-3c and PBEh-3c), r^2^SCAN-3c employs a more modern meta-GGA functional and the newer D4 dispersion correction, whereas PBEh-3c uses the D3 model. In addition, r^2^SCAN-3c is combined with a more flexible basis set optimized for noncovalent interactions. Nevertheless, both methods are well suited for the analysis of thermodynamic parameters of systems such as DIMEB and its complexes with fluorinated compounds.

The molar conductivity of the investigated system containing DIMEB and BFS was determined as a function of temperature and concentration. These data are presented in Table S15. The obtained data enabled a quantitative analysis of the complexation process in solution. Molar concentrations were calculated according to the relationships described in [22], ensuring consistency with methodologies commonly applied to cyclodextrin-based systems. The values of the formation constant K_f_ [dm^3^/mol] and the theoretical molar conductivity Λ_CD(BFS)_ [S·cm^2^·mol^−1^] for the DIMEB:BFS system are presented in Table 1.

Based on conductometric measurements, the formation constants K_f_ of the complexes were determined using models reported in the literature [22,23,24]. The obtained values allow for a quantitative characterization of host–guest interactions and serve as a basis for the determination of thermodynamic functions of the complexation process.

(1) $$K_{f}=\frac{\left(1-\alpha \right)}{\alpha [C_{CD}-\left(1-\alpha \right)C_{BFS}]}$$

The observed molar conductivity of the solution can be described as:

(2) $$\Lambda_{obs}=\alpha \cdot \Lambda_{m}+(1-\alpha ){\cdot \Lambda }_{c}$$

The complex formation constant can be expressed using the following equation:

(3) $$K_{f}=\frac{\left(\Lambda_{m}-\Lambda_{obs}\right)}{\left(\Lambda_{obs}-\Lambda_{c}\right)\cdot C_{CD}}$$

The concentration of cyclodextrin can be determined using the relationships described above, leading to the following expression:

(4) $$\left[C_{CD}\right]=C_{L}-\frac{C_{BFS}(\Lambda_{m}-\Lambda_{obs})}{\left(\Lambda_{m}-\Lambda_{c}\right)}$$

This leads to the following expression for the complexation constant:

(5) $$\Lambda =\left[K_{f}(c_{BFS}-C_{CD}-1+\sqrt{K_{f}^{2}(C_{CD}-C_{BFS}{)}^{2}+2K_{f}\left(C_{BFS}+C_{CD}\right)+1}\right]\cdot \left[\frac{\Lambda_{BFS}-\Lambda_{\left(BFS\right)CD}}{2K_{f}C_{BFS}}\right]+\Lambda_{(BFS)CD}$$

where terms are defined as follows:

C_L_—total concentration of cyclodextrin in the solution, which includes both free cyclodextrin molecules and those bound to BFS in the form of a complex;

Λ—molar conductivity of the solution prior to the addition of cyclodextrin;

Λ_(BFS)CD_—molar conductivity of the solution (BFS) with added cyclodextrin;

Λ_c_—molar conductivity of the complexed ion;

C_CD_—calculated current concentration of free cyclodextrin;

C_BFS_—concentration of BFS in solution.

Additionally, the values for Λ*_BFS_* and Λ*_(BFS)CD_* are described by the following dependencies:

(6) $$\Lambda_{BFS}=\Lambda_{0BFS}-S{c_{BFS}}^{\frac{1}{2}}+Ec_{BFS}lnc_{BFS}+J_{1}c_{S}+J_{2}{c_{BFS}}^{\frac{3}{2}}$$

(7) $$\Lambda_{(BFS)CD}=\Lambda_{0(BFS)CD}-S{c_{BFS}}^{\frac{1}{2}}+Ec_{BFS}lnc_{BFS}+J_{1}c_{S}+J_{2}{c_{BFS}}^{\frac{3}{2}}$$

The observed changes in molar conductivity indicate the formation of an inclusion complex between BFS and DIMEB. This process leads to a decrease in ionic mobility in solution, which is associated with partial encapsulation of the guest molecule within the cyclodextrin cavity, as well as reorganization of the solvation structure. The increased hydrophobicity of the DIMEB cavity, resulting from the presence of methyl groups, promotes stabilization of the complex through dispersion interactions and hydrogen bonding.

The temperature dependence of the formation constants indicates that the complexation process is predominantly enthalpy driven. The observed decrease in K_f_ values with increasing temperature suggests that complex stability arises mainly from energetically favorable interactions, typical of supramolecular systems.

Based on the K_f_ values, the thermodynamic functions of the complexation process were determined. The Gibbs free energy was calculated according to the following relationship:

(8) $$∆G\left(T\right)=-RTlnK_{f}(T)$$

The above relationship can be presented as follows.

(9) $$∆G\left(T\right)=A+BT+CT^{2}$$

The enthalpy values are presented as the first derivative of the free enthalpy after temperature at constant pressure.

(10) $$∆S^{o}=-(\frac{\partial ∆G^{o}}{\partial T}{)}_{p}=-B-2CT$$

The enthalpy is calculated from the following relationship:

(11) $$∆H^{o}=∆G^{o}+T∆S^{o}=A-CT^{2}$$

Additionally, polynomial approximation was applied to determine entropy and enthalpy changes from the temperature dependence.

The calculated thermodynamic parameters for the DIMEB:BFS complex show a clear dependence on temperature. Within the studied temperature range (293.15–313.15 K), the enthalpy values (ΔH) are negative and decrease with increasing temperature (from −84.10 to −103.90 kJ/mol), indicating the exothermic nature of the process.

At the same time, the Gibbs free energy values remain negative across the entire temperature range (from −13.26 to −7.67 kJ/mol), confirming the spontaneous nature of the complexation process. However, the absolute values of ΔG decrease with increasing temperature, suggesting a gradual reduction in complex stability.

The entropy values (ΔS; see Table S16) are negative (from −0.24 to −0.31 kJ/mol·K), indicating increased ordering of the system during complex formation. This effect can be attributed to the restricted mobility of the BFS molecule upon inclusion into the cyclodextrin cavity, as well as to the reorganization of the solvent structure.

The observed relationship between ΔH and ΔS indicates that the complexation process is governed primarily by enthalpic contributions, accompanied by an unfavorable entropic term. Such behavior is characteristic of supramolecular systems stabilized by non-covalent interactions.

The experimental results are in qualitative agreement with the DFT calculations performed for the DIMEB:BFS complex, as in both cases negative values of ΔH and ΔG are obtained, indicating a spontaneous and exothermic complexation process. However, most of the applied DFT methods systematically overestimate the thermodynamic parameters, with the exception of the composite methods r^2^SCAN-3c and PBEh-3c, which provide results closest to the experimental data, as discussed above.

## 3. Materials and Methods

### 3.1. Computational Analysis

In the configurational search performed to find the most stable complexes of DIMEB:BFS, we consider three initial configurations, O1, O2 and O3 (see Figure S1). In each configuration a different position of BFS towards DIMEB was considered. However, each configuration corresponds to an inclusion geometry, which—as shown in ref. [20]—results in significantly greater stability of the complexes. The geometries of both molecules DIMEB and BFS refer to its crystllographic coordinates taken from the Cambridge Structural Database (CSD; refcode 164185 [25] and 2423336 [19], for DIMEB and BFS, respectively). The initial configurations (O1, O2 and O3) were built in a HyperChem program (version 8.0) [26]. In the case of the crystallographic geometry of DIMEB, the water molecules were removed, while hydrogen atoms were added in HyperChem. Then for each configuration a systematic rotation of BFS around the X, Y, and Z axes was performed in 20° increments, resulting in 1457 structures per configuration. All generated structures were optimized using the universal force field (UFF) in Open Babel 3.1.1 [27]. Next, all structures were reoptimized at the PM7 [28] theory level in vacuo using the MOPAC2016 program [29]. The resulting complexes were ranked by their heats of formation (H_f_), and the six lowest-energy structures from each orientation set (O1–O3) were selected for further analysis.

Final optimizations were carried out at the DFT level using the ωB97X-D4/6-31G(d,p) method where ωB97X is a range-separated hybrid functional [30], D4 is Grimme’s empirical dispersion correction [31] and 6-31G(d,p) is the Pople basis set [32]. The calculations were performed in the ORCA 6.1 program [33] in the water modeled by the conductor-like polarizable continuum model (CPCM) [34]. For all structures, the vibrational frequency calculations were performed to obtain the thermochemistry parameter values and to check whether complexes correspond to true minima on the potential energy surface (PES). The absence of imaginary frequency confirms that the obtained complexes are minimum on the PES.

The stability of the complexes was estimated by calculating the complexation (ΔE_compl_) energy using these equations:

(12) $${∆E}_{compl}=E_{complex}^{OPT}-\left(E_{DIMEB}^{OPT}+E_{BFS}^{OPT}\right)$$

where terms are defined as follows:

$E_{complex}^{OPT}$—energy of the optimized complex;

$E_{DIMEB}^{OPT}$ and $E_{BFS}^{OPT}$—energies of DIMEB and BFS, respectively, in their most stable geometries.

The complexation energies were corrected for basis set superposition error (BSSE) using the counterpoise (CP) method [35].

For the five most stable complexes selected at the ωB97X-D4/6-31G(d,p) theory level, the reoptimizations and vibrational frequencies were performed at different theory levels to obtain the enthalpy and Gibbs energies. The following calculations were performed:

M06-2X-D3/6-31G(d,p) (PCM) [36,37,38], M05-2X-D3/6-31G(d,p) (PCM) [39], M08-HX-D3/6-31G(d,p) [40] (PCM), MN15-L-D3/6-31G(d,p) [41] (PCM), BHandHLYP-D4/6-31G(d,p) [42] (CPCM), CAM-B3LYP-D4/6-31G(d,p) [43] (CPCM), r^2^SCAN50-D4/6-31G(d,p) [44] (CPCM), ωB97X-D4/6-31G(d,p) (CPCM), B3LYP-D4/6-31G(d,p) [45] (CPCM), r^2^SCAN-3c (CPCM) [46] and PBEh-3c (CPCM) [47]. The r^2^SCAN-3c and PBEh-3c are the composite methods. The calculations performed with the CPCM of solvent were conducted in the ORCA 6.1 program, while with the PCM model were made in Gaussian16 (Rev. C02) [48]. D3 dispersion corrections are not natively implemented for M08-HX in Gaussian16; the IOp keywords (3/174–176) were used following the guidance in ref. [49], with S6 and SR6 set to 1.0000 and 1.6247, respectively. The same applies to MN15-L; however, in this case, the values are as follows: S6 = 1.0 while SR6 = 3.33388.

Non-covalent interaction (NCI) analyses were carried out using NCIPLOT (version 4.3) [50]. The three-dimensional visualization of NCI was generated with VMD (version 1.9.4) [51], whereas two-dimensional NCI plots were produced using Multiwfn (version 3.8) [52] in combination with gnuplot (version 6.0) [53].

### 3.2. Electrical Conductivity Estimation—Exploratory Method

An exploratory conductometric study was undertaken to characterize the electrical properties of the investigated systems. All measurements were performed under rigorously controlled thermal conditions within the range 293.15–313.15 K, applying 5 K increments. Temperature stabilization was achieved using a BU 20F thermostat (Lauda, Germany) in combination with a DLK 25 recirculating unit, ensuring fluctuations remained within ±0.005 K. Temperature values were independently monitored with an Amarell 3000TH digital thermometer.

Solutions were prepared by weight using a Sartorius RC 210D (Germany) analytical balance (±1 × 10^−5^ g), allowing for precise composition control. The experimental workflow followed a conductometric methodology consistent with approaches previously reported for electrolyte systems [54,55].

Electrical measurements were conducted using a Wayne-Kerr 6430B RLC bridge (UK) (instrumental uncertainty: 0.02%). A three-electrode conductivity cell fabricated from sodium-free glass was employed. Prior to data acquisition, the system was standardized using a potassium chloride reference solution of ultra-high purity (Merck, 99.999% (Germany)), enabling accurate determination of the cell constant.

Frequency-dependent measurements were carried out over the interval 0.2–20 kHz. The analysis included selected discrete frequencies spanning this range. The combined measurement uncertainty, incorporating contributions from instrumental performance, calibration procedures, and reagent purity, was estimated at approximately ±0.05%. A detailed uncertainty budget is provided in Table S15, while extended methodological considerations are discussed elsewhere [56].

### 3.3. Materials

BFS was obtained according to the procedure described in [19], while DIMEB was purchased from Merck with the high purity (≥98.0%).

### 3.4. Synthesis of the DIMEB:BFS Complex

For complex formation, DIMEB and BFS were mixed in water (1 mL) at a 1:1 molar ratio and stirred on a magnetic stirrer for 24 h at room temperature under the argon atmosphere.

## 4. Conclusions

In this work, we have analyzed the complex of the heptakis(2,6-di-O-methyl)-β-cyclodextrin (DIMEB) with the recently synthesized fluorinated compound butane-1,4-diyl bis(2,2,2-trifluoroethane-1-sulfonate) (BFS) as a model system for the analysis of thermodynamic parameters, with particular attention to enthalpy (ΔH) and Gibbs free energy (ΔG). The study employed two approaches: computational methods and conductometric measurements over the temperature range 293.15–313.15 K. The main conclusions are as follows:

Thermodynamic analysis should be performed on energetically favorable structures; therefore, an initial search for the most stable geometries was conducted. The theoretical energetic and structural results indicate that DIMEB forms a highly stable inclusion complex with BFS, in which the guest molecule primarily forms hydrogen bonds with the hydroxyl groups of DIMEB. Furthermore, a correlation between the number of hydrogen bonds and the deformation energy shows that the highest deformation energy of BFS is observed for the K2 complex, where the largest number of hydrogen bonds is formed.

Non-covalent interaction analysis reveals that the complex is predominantly stabilized by van der Waals dispersion forces. Additionally, strong intramolecular attractive interactions are observed, corresponding to hydrogen bonding between the hydroxyl groups of DIMEB.

Thermodynamic parameters (enthalpy (ΔH) and Gibbs energy (ΔG)) were calculated using the following methods: M06-2X-D3/6-31G(d,p) (PCM), M05-2X-D3/6-31G(d,p) (PCM), M08-HX-D3/6-31G(d,p) (PCM), MN15-L-D3/6-31G(d,p) (PCM), BHandHLYP-D4/6-31G(d,p) (CPCM), CAM-B3LYP-D4/6-31G(d,p) (CPCM), r^2^SCAN50-D4/6-31G(d,p) (CPCM), ωB97X-D4/6-31G(d,p) (CPCM), B3LYP-D4/6-31G(d,p) (CPCM), r^2^SCAN-3c (CPCM), and PBEh-3c (CPCM). Conventional DFT calculations significantly overestimate ΔH and ΔG, with deviations on the order of several tens of kJ/mol. The best agreement with experimental data was obtained for the composite methods r^2^SCAN-3c (CPCM) and PBEh-3c (CPCM). In the case of Gibbs free energy, r^2^SCAN-3c reproduces the experimental values most accurately, as confirmed by the coefficient of determination (R^2^ = 0.9972).

The observed changes in molar conductivity confirm the formation of an inclusion complex between BFS and DIMEB, resulting in reduced ionic mobility due to partial encapsulation of the guest molecule within the cyclodextrin cavity and accompanying reorganization of the solvation structure.

Conductometric analysis suggests that the complexation process is spontaneous. The thermodynamic profile indicates that the process is driven mainly by favorable enthalpic effects associated with stabilizing interactions, while the negative entropy change may result from an increase in system order during complex formation.

## Figures and Tables

**Table 1:** The value of constant formation K_f_ [dm^3^/mol], theoretical conductivity Λ_CD(BFS)_ [S∙cm^2^·mol^−1^] for containing BFS with the DIMEB.

T [K]	K_f_ [dm^3^/mol]	lnK_f_ [dm^3^/mol]	Λ_CD(DTIC)_ [S∙cm^2^·mol^−1^]	σ(Λ)
293.15	231 ± 8	5.442	82.21 ± 0.01	0.01
298.15	119 ± 7	4.779	95.55 ± 0.01	0.02
303.15	68 ± 4	4.220	110.12 ± 0.02	0.01
308.15	38 ± 2	3.638	124.40 ± 0.02	0.01
313.15	19 ± 3	2.994	142.31 ± 0.02	0.01

## Data Availability

The original contributions presented in this study are included in the article and Supplementary Materials. Further inquiries can be directed to the corresponding authors.

## Supplementary Materials

The following supporting information can be downloaded at: https://www.mdpi.com/article/10.3390/molecules31091420/s1, Figures S1 and S2; Tables S1–S16. Initial model of complexes; Complexation, interaction, deformation energies; Total energy values and coordinates of the most stable complexes; Enthalpy and Gibbs energies obtained from the theoretical calculations; Molar concentrations of complexes; Entropy values of complexes.
