# Supplementary material for: Probing DFT Functionals in the Analysis of Enthalpy and Gibbs Free Energy: A Case Study of a Heptakis(2,6-di-O-methyl)-β-cyclodextrin Complex with a Novel Fluorinated Compound
Source: Molecules. 2026 Apr 25;31(9):1420. doi: 10.3390/molecules31091420 (PMC13164785; doi:10.3390/molecules31091420)
Supplement: Supplementary file 1 [file molecules-31-01420-s001.zip › molecules-4261774-supplementary.pdf]

Electronic Supplementary Information

Probing DFT Functionals in the Analysis of Enthalpy and Gibbs Free Energy: A Case Study of a Heptakis(2,6-di-O-methyl)-β-Cyclodextrin Complex with a Novel Fluorinated Compound

Marta Hoelm*, Zdzisław Kinart*

Department of Physical Chemistry, Faculty of Chemistry, University of Lodz, Pomorska 163/165, Lodz, 90-236, Poland

* Correspondence: marta.hoelm@chemia.uni.lodz.pl (M.H.); zdzislaw.kinart@chemia.uni.lodz.pl (Z.K.)

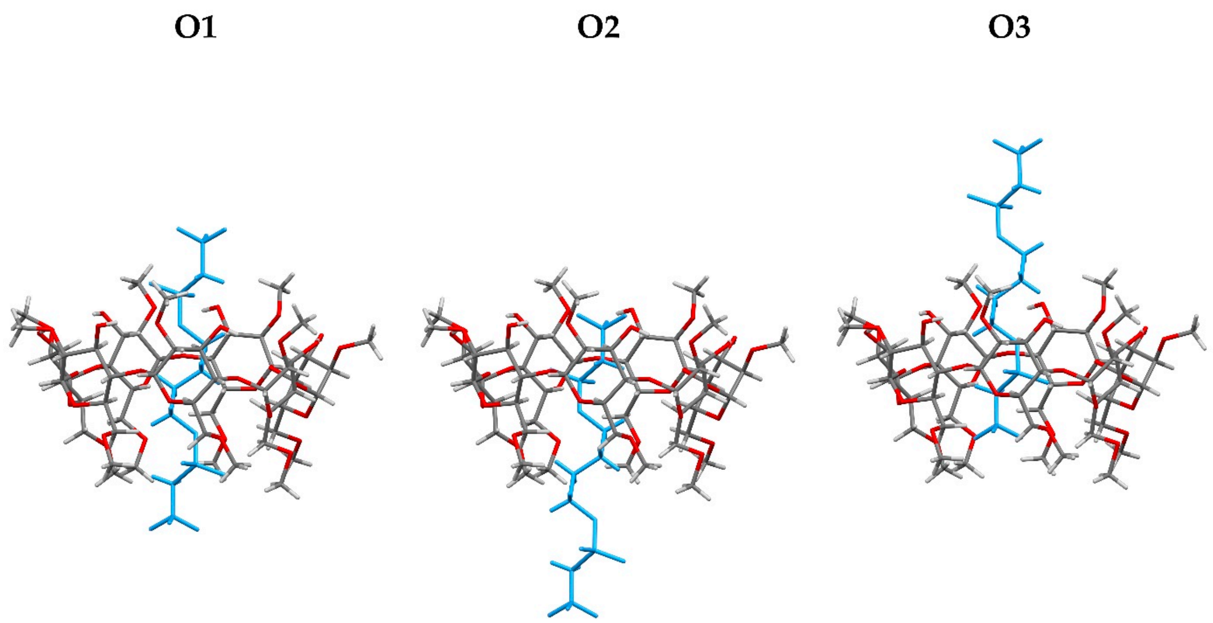

Figure S1. Initial models of the complexes used as starting points for the configurational search. Atom color scheme: carbon – dark grey, oxygen – red, hydrogen – light grey, BFS – blue.

Table S1. Complexation energy (ΔE_{compl}), interaction energy (ΔE_{int}), and deformation energies (ΔE_{def}), including contributions from BFS (ΔE_{def_BFS}), DIMEB (ΔE_{def_DIMEB}), and the total deformation energy of the complex (ΔE_{def_TOT}), as well as the number of hydrogen bonds (HB) formed in the most stable DIMEB:BFS complexes (K1–K5), calculated at the ωB97X-D4/6-31G(d,p) level of theory. Energy values are given in kJ/mol.

Complex	ΔE_{compl}	ΔE_{int}	ΔE_{def_BFS}	ΔE_{def_DIMEB}	ΔE_{def_TOT}	Number of HB
**K1**	–119.42	-117.87	0.63	-2.18	-1.55	8
**K2**	–101.99	-126.78	15.38	9.41	24.79	9
**K3**	–98.61	-122.24	8.86	14.77	23.63	8
**K4**	–96.35	-116.50	7.56	12.59	20.16	8
**K5**	–94.86	-101.92	2.08	4.97	7.06	6

Table S2. The total energy values [hartree] of DIMEB:BFS complexes obtained at the ωB97X-D4/6-31G(d,p) theory level.

Complex	Energy
K1	-6983.215
K2	-6983.202
K3	-6983.201
K4	-6983.203
K5	-6983.202

Table S3. The cartesian coordinates of DIMEB:BFS complexes obtained at the ωB97X-D4/6-31G(d,p) theory level.

Atom	K1			K2			K3			K4			K5		
	x	y	z	x	y	z	x	y	z	x	y	z	x	y	z
C	-5.0204	0.3411	3.0650	-4.7706	1.4600	2.4404	-4.5132	1.2212	2.4583	-4.3406	1.0035	2.6333	-4.4597	1.3411	2.4639
H	-5.9073	0.3565	3.7135	-5.6450	1.4141	3.1008	-5.2649	1.0313	3.2343	-5.2342	1.0552	3.2683	-5.3231	1.1827	3.1225
C	-5.0015	-0.9838	2.3154	-4.8728	0.3542	1.3920	-4.4550	0.0239	1.5097	-4.4656	-0.1857	1.6823	-4.3231	0.1365	1.5368
H	-3.9983	-1.1583	1.8990	-3.9609	0.3559	0.7785	-3.5979	0.1442	0.8380	-3.5704	-0.2337	1.0508	-3.4421	0.2679	0.8934
O	-5.9676	-1.0043	1.2583	-6.0083	0.6140	0.5630	-5.6616	-0.0414	0.7421	-5.6251	0.0108	0.8647	-5.5042	0.0564	0.7351
C	-5.8824	0.0535	0.3508	-5.8924	1.8178	-0.1620	-5.8770	1.1099	-0.0321	-5.5398	1.1423	0.0390	-5.7046	1.1736	-0.0911
H	-6.6774	-0.0968	-0.3895	-6.7766	1.8594	-0.8072	-6.7468	0.8966	-0.6624	-6.4856	1.1838	-0.5121	-6.6240	0.9717	-0.6495
C	-6.1003	1.3907	1.0854	-5.8416	3.0150	0.8039	-6.1038	2.3251	0.8810	-5.3734	2.4222	0.8709	-5.8585	2.4569	0.7371
H	-7.0980	1.3433	1.5449	-6.7919	3.0575	1.3567	-6.9752	2.1414	1.5251	-6.2960	2.5676	1.4512	-6.7743	2.3692	1.3407
C	-5.0445	1.5627	2.1626	-4.7025	2.8311	1.7886	-4.8599	2.5148	1.7294	-4.2020	2.2965	1.8384	-4.6685	2.6266	1.6704
H	-4.0681	1.6387	1.6632	-3.7570	2.8877	1.2273	-4.0283	2.7419	1.0457	-3.2690	2.2218	1.2551	-3.7684	2.8086	1.0600
C	-4.8861	-3.2806	2.9240	-5.0069	-1.0231	2.0030	-4.2789	-1.2899	2.2357	-4.5906	-1.5084	2.3999	-4.1558	-1.1705	2.2771
H	-5.4270	-3.6713	2.0512	-5.2360	-1.7540	1.2108	-4.3332	-2.1164	1.5085	-4.7858	-2.3090	1.6679	-4.1691	-2.0028	1.5546
H	-3.8157	-3.2423	2.6825	-4.0390	-1.2936	2.4503	-3.2757	-1.3016	2.6839	-3.6301	-1.7276	2.8826	-3.1725	-1.1653	2.7677
O	-5.3440	-1.9624	3.2272	-6.0152	-1.0186	2.9899	-5.2702	-1.4277	3.2288	-5.6225	-1.4375	3.3583	-5.1863	-1.3108	3.2283
C	-5.1216	-4.1381	4.1488	-6.0010	-2.2074	3.7533	-4.9863	-2.4969	4.1060	-5.6403	-2.5820	4.1855	-4.9535	-2.4038	4.0914
H	-6.1851	-4.1725	4.4022	-6.8029	-2.1340	4.4912	-5.8049	-2.5579	4.8270	-6.4675	-2.4668	4.8899	-5.7955	-2.4605	4.7851
H	-4.5714	-3.7265	5.0010	-5.0390	-2.3262	4.2723	-4.0429	-2.3221	4.6432	-4.6979	-2.6733	4.7435	-4.0239	-2.2607	4.6604
H	-4.7735	-5.1587	3.9668	-6.1735	-3.0912	3.1223	-4.9137	-3.4522	3.5660	-5.7961	-3.4984	3.5974	-4.8857	-3.3487	3.5328
O	-6.0628	2.4502	0.1419	-5.6189	4.2339	0.1232	-6.3015	3.5016	0.1172	-5.2073	3.4940	-0.0437	-5.9952	3.5173	-0.1885
C	-6.9399	3.5343	0.4348	-6.7807	4.7952	-0.4686	-7.6397	3.6977	-0.3236	-5.7096	4.7508	0.4035	-6.6713	4.6709	0.2950
H	-6.8291	4.2544	-0.3780	-6.4907	5.7760	-0.8500	-7.6571	4.6548	-0.8472	-5.5438	5.4565	-0.4116	-6.8377	5.3188	-0.5682
H	-6.6783	4.0022	1.3876	-7.5785	4.9125	0.2754	-8.3237	3.7367	0.5324	-5.1781	5.0818	1.2992	-6.0731	5.1939	1.0461
H	-7.9787	3.1838	0.4705	-7.1499	4.1837	-1.3017	-7.9628	2.9036	-1.0081	-6.7833	4.6820	0.6181	-7.6400	4.3955	0.7318
O	-5.3103	2.7212	2.9261	-4.7710	3.8571	2.7583	-5.0585	3.5777	2.6363	-4.1707	3.4187	2.6910	-4.9041	3.7055	2.5474
H	-4.7481	2.6807	3.7204	-4.2378	3.5829	3.5287	-4.2781	3.6114	3.2234	-3.6001	3.2003	3.4502	-4.1446	3.7493	3.1542
O	-4.6148	-0.0161	-0.2904	-4.7188	1.8424	-0.9245	-4.7522	1.4084	-0.8187	-4.4584	1.0601	-0.8535	-4.6256	1.3586	-0.9721
C	-4.5172	0.6508	-1.5456	-4.8129	1.7363	-2.3338	-4.8320	1.3228	-2.2284	-4.7363	0.6736	-2.1981	-4.8379	1.1045	-2.3535
H	-5.4122	1.2550	-1.7207	-5.8524	1.8730	-2.6580	-5.8793	1.2629	-2.5513	-5.7948	0.4069	-2.3043	-5.9115	1.0276	-2.5649
C	-4.4038	-0.3621	-2.6878	-4.3190	0.3707	-2.8079	-4.0805	0.0959	-2.7354	-3.8846	-0.5357	-2.5756	-4.1542	-0.1942	-2.7752
H	-3.4972	-0.9677	-2.5482	-3.2892	0.2187	-2.4526	-3.0365	0.1371	-2.3929	-2.8274	-0.3176	-2.3780	-3.0872	-0.1343	-2.5346
O	-4.3156	0.3461	-3.9253	-4.3431	0.3460	-4.2373	-4.1201	0.1106	-4.1637	-4.0764	-0.8038	-3.9691	-4.3078	-0.3543	-4.1883
C	-3.1786	1.1654	-4.0260	-3.5041	1.3067	-4.8288	-3.4586	1.2194	-4.7185	-3.6410	0.2400	-4.8003	-3.6813	0.6651	-4.9252
H	-3.2467	1.6566	-5.0021	-3.6144	1.1765	-5.9109	-3.5331	1.1033	-5.8044	-3.7665	-0.1126	-5.8293	-3.8311	0.4138	-5.9803
C	-3.1637	2.2175	-2.9090	-3.9253	2.7273	-4.4282	-4.1202	2.5346	-4.2829	-4.4619	1.5132	-4.5564	-4.3095	2.0267	-4.6083
H	-4.0247	2.8835	-3.0665	-4.9342	2.9046	-4.8281	-5.1369	2.5581	-4.7012	-5.5039	1.3294	-4.8593	-5.3583	2.0021	-4.9375
C	-3.2896	1.5593	-1.5340	-3.9572	2.8571	-2.9134	-4.2104	2.6080	-2.7644	-4.4222	1.8810	-3.0785	-4.2695	2.3006	-3.1103
H	-2.3975	0.9413	-1.3602	-2.9316	2.7441	-2.5312	-3.1878	2.6889	-2.3600	-3.3928	2.1945	-2.8555	-3.2146	2.4230	-2.8113
C	-5.5914	-1.2961	-2.7620	-5.1618	-0.7734	-2.2903	-4.6665	-1.2038	-2.2320	-4.2471	-1.7863	-1.8104	-4.7090	-1.4149	-2.0767
H	-5.4884	-1.9462	-3.6453	-4.8445	-1.7103	-2.7762	-4.1869	-2.0474	-2.7545	-3.6210	-2.6214	-2.1624	-4.2920	-2.3223	-2.5433
H	-5.5923	-1.9401	-1.8688	-4.9841	-0.8741	-1.2104	-4.4352	-1.2913	-1.1597	-4.0190	-1.6247	-0.7463	-4.3797	-1.3911	-1.0280
O	-6.7803	-0.5416	-2.8316	-6.5248	-0.5147	-2.5400	-6.0616	-1.2127	-2.4348	-5.6143	-2.0735	-1.9962	-6.1166	-1.4126	-2.1499
C	-7.9245	-1.3705	-2.8475	-7.3584	-1.4528	-1.8917	-6.6751	-2.3063	-1.7837	-6.0415	-3.1438	-1.1826	-6.6807	-2.4127	-1.3269
H	-8.8003	-0.7207	-2.9040	-8.3924	-1.1967	-2.1338	-7.7466	-2.2517	-1.9882	-7.0832	-3.3547	-1.4342	-7.7657	-2.3595	-1.4416
H	-7.9848	-1.9802	-1.9340	-7.2190	-1.4098	-0.8022	-6.5090	-2.2558	-0.6981	-5.9758	-2.8809	-0.1167	-6.4176	-2.2423	-0.2733
H	-7.9163	-2.0430	-3.7171	-7.1526	-2.4769	-2.2358	-6.2862	-3.2644	-2.1577	-5.4388	-4.0471	-1.3570	-6.3378	-3.4149	-1.6223
O	-1.9576	2.9581	-3.0261	-3.0069	3.6294	-5.0201	-3.3467	3.5853	-4.8305	-3.8831	2.4996	-5.3906	-3.5936	2.9976	-5.3487
C	-2.0564	4.3330	-2.6581	-3.5700	4.8733	-5.4200	-4.0763	4.7618	-5.1549	-4.6686	3.6615	-5.6224	-4.3651	4.1066	-5.7918
H	-1.0612	4.7607	-2.8019	-2.7708	5.4380	-5.9042	-3.3705	5.4474	-5.6280	-4.1894	4.1995	-6.4435	-3.7014	4.7286	-6.3959
H	-2.3572	4.4291	-1.6124	-3.9435	5.4254	-4.5531	-4.4985	5.2241	-4.2579	-4.7112	4.2964	-4.7339	-4.7461	4.6833	-4.9443
H	-2.7762	4.8547	-3.3012	-4.3882	4.7185	-6.1352	-4.8861	4.5334	-5.8600	-5.6894	3.3861	-5.9169	-5.2069	3.7702	-6.4107
O	-3.3280	2.5192	-0.4991	-4.4791	4.1169	-2.5554	-4.9841	3.7293	-2.3819	-5.3439	2.9233	-2.8299	-5.0098	3.4679	-2.8354
H	-4.2597	2.7563	-0.3433	-4.4851	4.1526	-1.5835	-5.2968	3.5745	-1.4703	-5.2911	3.1515	-1.8845	-5.1626	3.5040	-1.8737
O	-2.0013	0.4085	-3.9375	-2.1678	1.1300	-4.4436	-2.1135	1.2691	-4.3196	-2.2951	0.5405	-4.5388	-2.3102	0.7482	-4.6284
C	-1.2698	0.1888	-5.1388	-1.2784	0.5212	-5.3698	-1.1166	0.9192	-5.2680	-1.3370	0.3895	-5.5723	-1.3750	0.3154	-5.6054
H	-1.9163	0.3460	-6.0115	-1.8179	0.2250	-6.2781	-1.5516	0.8714	-6.2740	-1.8235	0.4189	-6.5552	-1.8784	0.1579	-6.5672
C	-0.8006	-1.2669	-5.1339	-0.6480	-0.7237	-4.7474	-0.4923	-0.4342	-4.9358	-0.5898	-0.9353	-5.4177	-0.7118	-0.9932	-5.1771

H	-0.3355	-1.4898	-4.1630	-0.1712	-0.4614	-3.7928	-0.0819	-0.4082	-3.9173	-0.1796	-0.9980	-4.3991	-0.2609	-0.8676	-4.1813
O	0.1460	-1.4857	-6.1818	0.3315	-1.2419	-5.6541	0.5594	-0.6881	-5.8730	0.4779	-0.9878	-6.3685	0.3029	-1.3270	-6.1292
C	1.2746	-0.6497	-6.1097	1.3868	-0.3564	-5.9197	1.6041	0.2470	-5.8029	1.4310	0.0322	-6.1935	1.3272	-0.3692	-6.2256
H	1.9433	-0.9651	-6.9179	2.0570	-0.8824	-6.6085	2.3316	-0.0574	-6.5631	2.1971	-0.1353	-6.9585	2.0282	-0.7534	-6.9747
C	0.8620	0.8153	-6.2894	0.8680	0.9262	-6.5782	1.1034	1.6620	-6.1148	0.7860	1.4087	-6.3886	0.7684	0.9833	-6.6797
H	0.3849	0.9215	-7.2740	0.4447	0.6529	-7.5554	0.7708	1.6685	-7.1627	0.4324	1.4682	-7.4278	0.3647	0.8552	-7.6941
C	-0.1164	1.1897	-5.1884	-0.2212	1.5625	-5.7287	-0.0765	2.0336	-5.2290	-0.3962	1.5817	-5.4468	-0.3539	1.4374	-5.7579
H	0.4299	1.1336	-4.2354	0.2381	1.9162	-4.7916	0.2878	2.1286	-4.1927	-0.0166	1.6042	-4.4143	0.0784	1.6448	-4.7649
C	-1.9462	-2.2363	-5.3370	-1.6466	-1.8275	-4.4801	-1.4756	-1.5809	-4.9977	-1.4838	-2.1381	-5.6319	-1.6863	-2.1484	-5.1042
H	-1.5556	-3.2668	-5.3498	-1.1124	-2.7125	-4.0960	-0.9263	-2.5324	-4.9024	-0.8644	-3.0477	-5.6820	-1.1326	-3.0802	-4.9001
H	-2.6352	-2.1493	-4.4847	-2.3463	-1.4916	-3.7005	-2.1585	-1.4982	-4.1419	-2.1578	-2.2269	-4.7664	-2.3731	-1.9723	-4.2673
O	-2.6104	-1.9370	-6.5432	-2.3332	-2.1359	-5.6707	-2.1913	-1.5330	-6.2109	-2.2289	-1.9817	-6.8199	-2.4048	-2.2418	-6.3134
C	-3.8158	-2.6590	-6.6701	-3.3566	-3.0834	-5.4587	-3.2486	-2.4691	-6.2269	-3.1995	-2.9981	-6.9624	-3.4872	-3.1429	-6.2045
H	-4.2656	-2.3848	-7.6268	-3.8197	-3.2902	-6.4261	-3.7518	-2.3810	-7.1926	-3.7320	-2.8157	-7.8984	-3.9950	-3.1658	-7.1713
H	-4.5154	-2.4084	-5.8580	-4.1210	-2.6946	-4.7701	-3.9673	-2.2584	-5.4225	-3.9158	-2.9775	-6.1281	-4.1937	-2.8113	-5.4301
H	-3.6388	-3.7442	-6.6538	-2.9565	-4.0201	-5.0441	-2.8753	-3.4970	-6.1101	-2.7340	-3.9935	-7.0002	-3.1413	-4.1571	-5.9572
O	2.0347	1.6102	-6.2268	1.9766	1.7875	-6.7554	2.1939	2.5512	-5.9468	1.7861	2.3865	-6.1614	1.8416	1.9078	-6.6989
C	2.0064	2.7844	-7.0321	1.9327	2.5920	-7.9270	2.2265	3.6290	-6.8762	1.6834	3.5455	-6.9799	1.7851	2.8636	-7.7514
H	2.9711	3.2774	-6.8958	2.8695	3.1521	-7.9579	3.1283	4.2042	-6.6575	2.5337	4.1830	-6.7292	2.6872	3.4732	-7.6697
H	1.1990	3.4488	-6.7140	1.0862	3.2837	-7.8935	1.3434	4.2643	-6.7632	0.7485	4.0779	-6.7831	0.8985	3.4964	-7.6529
H	1.8759	2.5269	-8.0910	1.8567	1.9656	-8.8251	2.2768	3.2538	-7.9064	1.7337	3.2766	-8.0430	1.7721	2.3658	-8.7295
O	-0.6015	2.4968	-5.4078	-0.7948	2.6311	-6.4470	-0.6289	3.2493	-5.6802	-1.0692	2.7833	-5.7573	-0.9523	2.5916	-6.3014
H	-1.0482	2.7700	-4.5852	-1.5079	3.0078	-5.8988	-1.4877	3.3680	-5.2359	-1.9676	2.7216	-5.3861	-1.7847	2.7528	-5.8213
O	1.9007	-0.7651	-4.8627	2.0708	-0.0053	-4.7434	2.2011	0.2549	-4.5312	1.9970	-0.0082	-4.9096	1.9731	-0.1823	-4.9946
C	3.1542	-1.4260	-4.7595	3.3654	-0.5483	-4.5287	3.4199	-0.4606	-4.3830	3.3156	-0.5157	-4.7549	3.2523	-0.7707	-4.8063
H	3.3658	-2.0056	-5.6698	3.7227	-1.0498	-5.4400	3.6695	-0.9819	-5.3184	3.7331	-0.7964	-5.7334	3.5642	-1.3061	-5.7150
C	3.0684	-2.3553	-3.5492	3.3705	-1.5547	-3.3750	3.3168	-1.4963	-3.2625	3.2969	-1.7440	-3.8407	3.2136	-1.7521	-3.6336
H	2.6570	-1.7856	-2.7084	2.9648	-1.0829	-2.4690	3.0184	-1.0039	-2.3256	2.7436	-1.4956	-2.9228	2.7913	-1.2488	-2.7516
O	4.3668	-2.8347	-3.1994	4.7209	-1.9654	-3.1493	4.5998	-2.1091	-3.1066	4.6368	-2.1276	-3.5140	4.5424	-2.1989	-3.3542
C	5.2529	-1.8037	-2.8556	5.5743	-0.9254	-2.7463	5.6172	-1.2199	-2.7258	5.4237	-1.1401	-2.9046	5.4392	-1.1780	-3.0001
H	6.1767	-2.2837	-2.5151	6.5560	-1.3839	-2.5857	6.5280	-1.8209	-2.6284	6.4139	-1.5893	-2.7716	6.3977	-1.6732	-2.8095
C	5.5297	-0.9092	-4.0662	5.6685	0.1510	-3.8305	5.8241	-0.1302	-3.7832	5.5369	0.0961	-3.8030	5.5900	-0.1712	-4.1446
H	6.0192	-1.5118	-4.8449	6.1537	-0.2956	-4.7102	6.1961	-0.6078	-4.7011	6.0651	-0.2022	-4.7202	6.0390	-0.6956	-5.0002
C	2.1720	-3.5485	-3.7881	2.5616	-2.7934	-3.6815	2.3273	-2.5927	-3.5837	2.6643	-2.9391	-4.5397	2.3946	-2.9816	-3.9536
H	1.3022	-3.2373	-4.3874	1.5629	-2.4920	-4.0272	1.3772	-2.1404	-3.8974	1.7986	-2.6034	-5.1270	1.4647	-2.6890	-4.4615
H	2.7195	-4.3189	-4.3535	3.0469	-3.3602	-4.4924	2.7093	-3.2005	-4.4189	3.4081	-3.3544	-5.2290	2.9710	-3.6212	-4.6404
C	4.2207	-0.3397	-4.5916	4.2820	0.6376	-4.2285	4.5081	0.5704	-4.0866	4.1570	0.6160	-4.1712	4.2302	0.3750	-4.5518
H	3.8445	0.3688	-3.8385	3.8504	1.1830	-3.3724	4.2034	1.1319	-3.1888	3.6620	0.9630	-3.2495	3.8371	0.9702	-3.7122
O	1.7548	-4.0446	-2.5339	2.4621	-3.5918	-2.5184	2.1365	-3.3993	-2.4384	2.2904	-3.9778	-3.6570	2.1061	-3.6796	-2.7581
C	0.8644	-5.1347	-2.6570	1.5926	-4.6906	-2.7213	1.1695	-4.4099	-2.6620	1.0135	-3.7754	-3.0851	1.4485	-4.9053	-3.0233
H	0.5395	-5.4007	-1.6488	1.5799	-5.2732	-1.7973	1.1138	-5.0111	-1.7514	0.7966	-4.6450	-2.4582	1.1971	-5.3582	-2.0613
H	1.3548	-5.9975	-3.1295	1.9443	-5.3267	-3.5447	1.4587	-5.0534	-3.5033	0.2399	-3.7002	-3.8621	2.1017	-5.5849	-3.5874
H	-0.0167	-4.8575	-3.2528	0.5723	-4.3476	-2.9471	0.1832	-3.9695	-2.8699	0.9712	-2.8673	-2.4658	0.5251	-4.7424	-3.5960
O	6.4088	0.1018	-3.6076	6.4711	1.1941	-3.3103	6.8007	0.7534	-3.2659	6.3003	1.0557	-3.0990	6.4599	0.8481	-3.6909
C	7.2243	0.7046	-4.6051	7.2764	1.8688	-4.2686	7.6331	1.3690	-4.2407	7.1312	1.8811	-3.9063	7.2954	1.4165	-4.6913
H	7.9055	1.3800	-4.0835	7.8705	2.6011	-3.7182	8.3672	1.9644	-3.6941	7.6992	2.5156	-3.2228	7.9442	2.1350	-4.1864
H	6.6197	1.2619	-5.3254	6.6570	2.3766	-5.0132	7.0508	2.0135	-4.9053	6.5317	2.5005	-4.5793	6.7002	1.9248	-5.4552
H	7.8080	-0.0587	-5.1353	7.9493	1.1641	-4.7737	8.1559	0.6098	-4.8365	7.8273	1.2697	-4.4947	7.9132	0.6429	-5.1653
O	4.4496	0.3174	-5.8192	4.4026	1.4736	-5.3553	4.6847	1.4312	-5.1884	4.2946	1.6654	-5.1004	4.3786	1.1652	-5.7089
H	3.6501	0.8428	-6.0094	3.5077	1.6492	-5.6993	3.8304	1.8664	-5.3662	3.4005	1.9429	-5.3732	3.4923	1.4738	-5.9736
O	4.6996	-1.0020	-1.8394	5.1161	-0.3185	-1.5669	5.2982	-0.5991	-1.5091	4.8950	-0.7540	-1.6579	5.0048	-0.4876	-1.8589
C	5.3855	-0.8759	-0.6062	5.8381	-0.5216	-0.3595	6.1256	-0.8325	-0.3771	5.6535	-1.0323	-0.4844	5.7156	-0.6658	-0.6405
H	6.4414	-1.1605	-0.7215	6.8420	-0.9141	-0.5785	7.1232	-1.1672	-0.6984	6.6825	-1.3109	-0.7552	6.6777	-1.1636	-0.8313
C	4.7386	-1.7293	0.4863	5.0993	-1.4929	0.5641	5.4985	-1.8898	0.5390	5.0081	-2.1661	0.3146	4.9044	-1.4932	0.3589
H	3.6856	-1.4283	0.5835	4.0594	-1.1590	0.6841	4.4339	-1.6489	0.6718	3.9541	-1.9095	0.4966	3.9124	-1.0419	0.4909
O	5.4256	-1.4792	1.7136	5.7546	-1.5080	1.8348	6.1525	-1.9012	1.8089	5.6868	-2.3199	1.5634	5.6020	-1.5067	1.6086
C	5.2921	-0.1588	2.1757	5.7494	-0.2639	2.4870	6.1339	-0.6670	2.4793	5.6349	-1.1883	2.3901	5.7477	-0.2389	2.1935
H	5.8573	-0.1173	3.1137	6.2301	-0.4275	3.4578	6.6266	-0.8375	3.4429	6.1932	-1.4497	3.2956	6.2664	-0.4014	3.1447
C	5.8821	0.8266	1.1525	6.5367	0.7704	1.6785	6.9001	0.3839	1.6724	6.2985	0.0080	1.7022	6.5834	0.6752	1.2930
H	6.9652	0.6448	1.0976	7.5848	0.4417	1.6361	7.9402	0.0436	1.5719	7.3639	-0.2333	1.5795	7.5972	0.2544	1.2305
C	5.2983	0.6002	-0.2315	5.9830	0.8660	0.2645	6.2753	0.5297	0.2944	5.6957	0.2617	0.3268	5.9876	0.7400	-0.1062

H	4.2308	0.8788	-0.2150	4.9755	1.3086	0.3324	5.2652	0.9469	0.4287	4.6561	0.6014	0.4650	5.0196	1.2626	-0.0394
C	4.8241	-3.2208	0.2101	5.1016	-2.9076	0.0306	5.6690	-3.2828	-0.0511	5.1004	-3.5097	-0.3877	4.7322	-2.9302	-0.0792
H	4.4626	-3.4130	-0.8110	4.8009	-2.8936	-1.0264	5.2783	-3.2816	-1.0794	4.8567	-3.3899	-1.4527	4.3740	-2.9495	-1.1184
H	5.8722	-3.5334	0.2785	6.1217	-3.3214	0.0960	6.7410	-3.5111	-0.0802	6.1298	-3.8749	-0.2999	5.7075	-3.4445	-0.0433
O	4.1049	-4.0072	1.1373	4.1956	-3.6936	0.7775	5.0580	-4.3058	0.7002	4.2560	-4.4784	0.2031	3.8008	-3.5668	0.7701
C	2.6986	-3.9363	0.9719	4.0883	-4.9966	0.2375	3.6599	-4.3933	0.4989	2.9375	-4.4020	-0.3034	3.4968	-4.8697	0.3168
H	2.2664	-4.8074	1.4709	3.3271	-5.5296	0.8113	3.3385	-5.3687	0.8734	2.3605	-5.2128	0.1494	2.7489	-5.2890	0.9949
H	2.4215	-3.9560	-0.0918	5.0427	-5.5380	0.3091	3.4022	-4.3111	-0.5670	2.9357	-4.5152	-1.3959	4.3874	-5.5155	0.3224
H	2.2753	-3.0349	1.4366	3.7826	-4.9558	-0.8176	3.1165	-3.6171	1.0536	2.4578	-3.4431	-0.0534	3.0895	-4.8426	-0.7038
O	5.6350	2.1687	1.5239	6.4391	2.0019	2.3696	6.8546	1.6010	2.3954	6.1518	1.1343	2.5503	6.6231	1.9451	1.9162
C	6.6116	2.7173	2.3994	7.5843	2.8387	2.2672	8.0258	2.4015	2.2908	7.2686	2.0176	2.5650	7.8224	2.6805	1.7113
H	6.3370	3.7622	2.5545	7.3875	3.7165	2.8857	7.8636	3.2783	2.9207	7.0411	2.7967	3.2954	7.7328	3.5960	2.2995
H	7.6097	2.6635	1.9483	7.7539	3.1449	1.2308	8.1914	2.7137	1.2557	7.4211	2.4642	1.5787	7.9553	2.9284	0.6544
H	6.6232	2.2021	3.3680	8.4768	2.3231	2.6447	8.9051	1.8531	2.6522	8.1796	1.4882	2.8718	8.6914	2.1092	2.0627
O	6.0069	1.4088	-1.1474	6.8353	1.6718	-0.5165	7.0744	1.3844	-0.4933	6.4657	1.2471	-0.3209	6.8796	1.4308	-0.9508
H	5.9152	1.0238	-2.0394	6.5572	1.5847	-1.4462	6.7994	1.2783	-1.4215	6.2253	1.2486	-1.2653	6.5777	1.3116	-1.8694
O	3.9474	0.1732	2.4028	4.4452	0.2236	2.6578	4.8254	-0.1994	2.6655	4.3127	-0.8496	2.7113	4.5043	0.3756	2.4097
C	3.5105	0.2848	3.7497	3.8537	0.1559	3.9469	4.2428	-0.2750	3.9592	3.8279	-1.1820	4.0066	4.0335	0.5122	3.7432
H	4.3777	0.3008	4.4263	4.6081	-0.1181	4.6989	4.9526	-0.7133	4.6756	4.5803	-1.7670	4.5555	4.8558	0.3458	4.4548
C	2.5856	-0.8648	4.1548	2.7097	-0.8570	3.9772	2.9714	-1.1209	3.9109	2.5449	-2.0050	3.8853	2.8954	-0.4646	4.0428
H	1.7299	-0.9036	3.4642	1.9967	-0.6129	3.1748	2.3134	-0.7316	3.1200	1.8397	-1.4887	3.2174	2.0980	-0.3279	3.2999
O	2.1294	-0.6263	5.4894	2.0665	-0.7797	5.2518	2.3126	-1.0424	5.1760	1.9682	-2.1615	5.1823	2.3975	-0.1915	5.3548
C	1.3951	0.5588	5.6611	1.4886	0.4641	5.5478	1.8973	0.2444	5.5416	1.6183	-0.9594	5.8154	1.8436	1.0881	5.5040
H	1.1572	0.6070	6.7296	1.0794	0.3647	6.5590	1.4147	0.1350	6.5179	1.1594	-1.2451	6.7670	1.5178	1.1526	6.5480
C	2.2318	1.7839	5.2664	2.5450	1.5771	5.5368	3.0999	1.1865	5.6536	2.8599	-0.0960	6.0565	2.8905	2.1724	5.2243
H	3.0737	1.8533	5.9704	3.2293	1.3906	6.3768	3.7224	0.8377	6.4901	3.5200	-0.6305	6.7551	3.6597	2.1029	6.0068
C	2.7852	1.6232	3.8582	3.3411	1.5627	4.2428	3.9293	1.1623	4.3787	3.5978	0.1371	4.7464	3.5531	1.9566	3.8710
H	1.9410	1.6168	3.1494	2.6567	1.8456	3.4256	3.3289	1.6145	3.5726	2.9586	0.7689	4.1094	2.7960	2.1257	3.0856
C	3.2795	-2.2087	4.1606	3.1773	-2.2864	3.8104	3.2275	-2.5918	3.6643	2.7990	-3.4025	3.3630	3.3381	-1.9097	4.0223
H	3.8613	-2.3395	3.2379	3.9669	-2.3541	3.0512	3.9957	-2.7349	2.8909	3.4664	-3.3824	2.4894	3.8933	-2.1124	3.0961
H	3.9740	-2.2462	5.0162	3.5841	-2.6344	4.7727	3.5957	-3.0371	4.6002	3.2880	-3.9839	4.1591	4.0097	-2.0970	4.8772
O	2.3018	-3.2226	4.2685	2.0698	-3.0822	3.4271	2.0040	-3.1928	3.2770	1.5550	-3.9920	3.0244	2.1954	-2.7353	4.0977
C	2.8882	-4.5014	4.4038	2.3597	-4.4650	3.5087	1.9250	-4.5594	3.6337	1.6159	-5.4061	2.9851	2.5372	-4.1049	4.0588
H	2.0761	-5.2321	4.4342	1.4956	-5.0017	3.1108	0.9422	-4.9173	3.3237	0.6613	-5.7607	2.5881	1.6062	-4.6763	4.0827
H	3.4718	-4.5735	5.3334	2.5273	-4.7693	4.5516	2.0335	-4.6870	4.7199	1.7686	-5.8184	3.9919	3.1540	-4.3876	4.9247
H	3.5490	-4.7243	3.5544	3.2499	-4.7129	2.9166	2.6998	-5.1539	3.1306	2.4292	-5.7541	2.3340	3.0916	-4.3440	3.1402
O	1.4075	2.9298	5.3867	1.8756	2.8127	5.7227	2.5935	2.4783	5.9318	2.4113	1.1129	6.6405	2.2332	3.4245	5.3004
C	2.0849	4.1028	5.8248	2.5923	3.7665	6.4997	3.4059	3.2735	6.7851	3.3245	1.7199	7.5448	3.0162	4.4862	5.8326
H	1.3274	4.8833	5.9209	1.9415	4.6370	6.6035	2.8656	4.2078	6.9511	2.8180	2.5912	7.9649	2.3536	5.3490	5.9152
H	2.8427	4.4068	5.0977	3.5215	4.0536	5.9999	4.3747	3.4811	6.3218	4.2378	2.0322	7.0305	3.8544	4.7215	5.1703
H	2.5598	3.9368	6.8003	2.8213	3.3644	7.4948	3.5633	2.7718	7.7487	3.5835	1.0277	8.3566	3.4003	4.2247	6.8269
O	3.6678	2.6853	3.5760	4.4116	2.4721	4.3453	5.1163	1.8903	4.5948	4.8287	0.7720	5.0106	4.6290	2.8567	3.7396
H	4.0746	2.4964	2.7125	4.9876	2.3438	3.5699	5.6493	1.8432	3.7799	5.2528	0.9588	4.1522	5.1729	2.5668	2.9851
O	0.2229	0.5548	4.8971	0.4702	0.7914	4.6419	0.9937	0.7805	4.6083	0.7211	-0.2114	5.0397	0.7580	1.2908	4.6398
C	-1.0104	0.3101	5.5677	-0.8704	0.6087	5.0856	-0.3627	0.9081	5.0074	-0.6251	-0.0996	5.4792	-0.5509	1.2250	5.1912
H	-0.8291	0.0739	6.6235	-0.8774	0.1952	6.1017	-0.4468	0.7980	6.0958	-0.7050	-0.3880	6.5346	-0.5002	1.0728	6.2765
C	-1.7098	-0.8791	4.9096	-1.6095	-0.3656	4.1690	-1.2599	-0.1387	4.3493	-1.5475	-0.9912	4.6516	-1.3272	0.0673	4.5630
H	-1.7472	-0.7241	3.8249	-1.5403	-0.0235	3.1276	-1.2363	-0.0222	3.2552	-1.4382	-0.7504	3.5860	-1.2764	0.1523	3.4688
O	-3.0400	-0.9926	5.4259	-2.9818	-0.4184	4.5763	-2.5903	0.0831	4.8307	-2.8969	-0.7371	5.0581	-2.6919	0.1303	4.9912
C	-3.8265	0.1421	5.1850	-3.6445	0.8132	4.4927	-3.1323	1.3175	4.4395	-3.3126	0.5786	4.7939	-3.3443	1.3165	4.6192
H	-4.8356	-0.0946	5.5378	-4.6843	0.6214	4.7769	-4.1265	1.3638	4.8972	-4.3639	0.6308	5.0957	-4.3834	1.2004	4.9460
C	-3.2659	1.3635	5.9194	-3.0087	1.8439	5.4304	-2.2833	2.4963	4.9410	-2.4788	1.5824	5.5998	-2.6768	2.5235	5.2984
H	-3.2990	1.1745	7.0020	-3.1259	1.4945	6.4663	-2.3882	2.5431	6.0348	-2.6842	1.4173	6.6674	-2.7606	2.4063	6.3881
C	-1.8268	1.5996	5.4806	-1.5277	1.9866	5.1111	-0.8053	2.3129	4.6137	-0.9918	1.3745	5.3422	-1.2137	2.5679	4.9049
H	-1.8496	1.9089	4.4248	-1.4380	2.4306	4.1056	-0.6702	2.4144	3.5236	-0.7876	1.6772	4.3037	-1.1738	2.7400	3.8204
C	-1.0059	-2.1934	5.1622	-1.0695	-1.7756	4.2339	-0.8648	-1.5622	4.6691	-1.2566	-2.4673	4.8161	-0.7872	-1.2921	4.9423
H	-1.6126	-3.0083	4.7344	-1.6887	-2.4245	3.5935	-1.6259	-2.2488	4.2626	-2.0879	-3.0473	4.3866	-1.4605	-2.0652	4.5349
H	-0.0354	-2.1902	4.6456	-0.0455	-1.7940	3.8377	0.0911	-1.7883	4.1768	-0.3447	-2.7129	4.2530	0.2026	-1.4319	4.4876
O	-0.8283	-2.3771	6.5504	-1.0994	-2.2234	5.5721	-0.7512	-1.7176	6.0674	-1.0858	-2.7831	6.1817	-0.7053	-1.3965	6.3478
C	-0.0478	-3.5214	6.8283	-0.4634	-3.4757	5.7192	-0.1858	-2.9672	6.4047	-0.6017	-4.1000	6.3460	-0.1203	-2.6208	6.7418
H	0.0699	-3.5844	7.9127	-0.5503	-3.7679	6.7684	-0.1559	-3.0319	7.4949	-0.4839	-4.2771	7.4174	-0.0504	-2.6168	7.8321

H	0.9397	-3.4480	6.3521	0.5964	-3.4125	5.4390	0.8345	-3.0518	6.0062	0.3706	-4.2214	5.8474	0.8821	-2.7362	6.3070
H	-0.5404	-4.4377	6.4692	-0.9429	-4.2437	5.0931	-0.7906	-3.7991	6.0136	-1.3052	-4.8394	5.9358	-0.7367	-3.4763	6.4262
O	-4.1132	2.4504	5.5874	-3.7225	3.0522	5.2443	-2.8223	3.6697	4.3540	-2.9152	2.8736	5.2160	-3.2827	3.7410	4.8966
C	-4.1987	3.4660	6.5778	-3.7787	3.9053	6.3804	-2.6704	4.8605	5.1208	-2.8018	3.8721	6.2215	-4.4235	4.1024	5.6680
H	-4.9189	4.2013	6.2132	-4.4084	4.7533	6.1038	-3.1512	5.6567	4.5491	-3.2404	4.7830	5.8091	-4.7614	5.0710	5.2950
H	-3.2276	3.9430	6.7384	-2.7811	4.2555	6.6590	-1.6133	5.0947	5.2677	-1.7552	4.0488	6.4853	-4.1591	4.1892	6.7284
H	-4.5592	3.0502	7.5280	-4.2315	3.3820	7.2325	-3.1621	4.7576	6.0966	-3.3577	3.5796	7.1218	-5.2342	3.3726	5.5532
O	-1.2465	2.6038	6.2857	-0.9193	2.8073	6.0832	-0.0602	3.2888	5.3062	-0.2431	2.1555	6.2483	-0.5771	3.6140	5.6025
H	-0.3471	2.7630	5.9443	0.0342	2.8462	5.8825	0.8716	3.0048	5.3186	0.6817	1.8524	6.2055	0.3781	3.5612	5.4098
O	-3.8347	0.4726	3.8228	-3.5739	1.3309	3.1891	-3.2326	1.4151	3.0459	-3.1729	0.9060	3.4352	-3.2831	1.5266	3.2335
S	3.0749	3.4514	-2.4678	0.4148	-1.2488	-0.0068	-0.5109	-1.1618	-0.3951	-0.5593	-1.7080	0.1659	0.1901	-1.0872	0.3773
O	3.9455	2.9308	-3.5049	-0.6305	-0.9661	0.9660	-1.5177	-0.8910	0.6212	-1.4248	-1.0720	1.1475	1.5056	-1.0270	0.9859
F	4.3176	4.7068	1.0832	0.7916	-5.1111	0.5867	0.7415	-4.6581	0.8109	-1.5142	-5.4234	0.9949	-1.2309	-4.0164	-1.8555
O	2.1287	4.5080	-2.7807	0.0811	-1.1497	-1.4212	-0.9715	-1.5067	-1.7335	-0.8873	-1.5329	-1.2422	-0.9926	-0.9067	1.2037
F	2.9431	3.0646	0.7350	-0.7207	-3.9779	-0.4837	-1.0669	-4.0085	-0.1854	-2.6253	-3.8786	-0.0516	-1.5522	-1.8781	-2.0211
O	2.2955	2.1974	-1.8695	1.7016	-0.3553	0.3101	0.5327	0.0394	-0.4829	0.9657	-1.3043	0.4197	0.1924	-0.0387	-0.8253
F	2.4218	5.0635	0.0968	-0.5458	-3.7993	1.6684	-0.7090	-3.4467	1.8747	-2.2164	-3.6773	2.0636	-2.2737	-2.9539	-0.2865
C	4.1371	3.9504	-1.1183	1.1721	-2.8178	0.3572	0.6233	-2.3922	0.2178	-0.3790	-3.4320	0.5789	0.0783	-2.6451	-0.4845
H	4.8934	3.1631	-0.9989	1.8374	-3.0426	-0.4839	1.3013	-2.6394	-0.6087	0.0711	-3.9294	-0.2823	0.8794	-2.6932	-1.2338
H	4.6154	4.8771	-1.4464	1.7402	-2.7163	1.2889	1.1756	-1.9702	1.0626	0.2909	-3.4995	1.4476	0.2411	-3.4202	0.2696
C	0.9348	2.3799	-1.3482	1.9828	0.8521	-0.4700	0.2250	1.1580	-1.3847	1.4542	-0.0354	-0.1415	-0.9198	0.8972	-1.0057
H	0.2589	2.5172	-2.1971	1.3076	0.8866	-1.3305	-0.6779	0.9251	-1.9595	0.6965	0.3669	-0.8172	-1.8147	0.4765	-0.5396
H	0.9066	3.2665	-0.7125	3.0071	0.7179	-0.8275	1.0738	1.2061	-2.0721	2.3373	-0.3110	-0.7263	-1.0640	0.9374	-2.0890
C	3.4461	4.1976	0.2037	0.1579	-3.9236	0.5338	-0.1149	-3.6297	0.6875	-1.6991	-4.0983	0.8999	-1.2578	-2.8746	-1.1564
C	0.6140	1.1231	-0.5723	1.8480	2.0667	0.4239	0.0611	2.4302	-0.5823	1.8369	0.9063	0.9894	-0.5760	2.2582	-0.4337
H	-0.3633	1.2708	-0.0971	2.2661	2.9181	-0.1279	-0.0862	3.2380	-1.3099	2.2733	1.7996	0.5216	-1.4588	2.8921	-0.5924
H	1.3519	0.9987	0.2315	2.4798	1.9197	1.3089	0.9912	2.6477	-0.0439	2.6406	0.4262	1.5590	0.2468	2.6995	-1.0083
S	-1.2960	-2.7423	0.6680	-1.5876	4.9485	0.5022	-2.3784	6.0575	0.5754	-0.7300	2.9422	-1.0492	-1.5136	5.7806	1.9044
O	-1.6763	-2.3764	2.0215	-2.2082	4.0459	-0.4552	-1.9556	7.0467	-0.3973	-0.9016	2.0205	-2.1561	-2.9004	6.0164	1.5593
F	-3.7131	-3.6284	-2.3190	-4.5815	7.4129	1.0713	-6.2746	6.5589	0.3317	-3.5439	5.5664	-1.9048	0.0846	6.5412	5.4486
O	-0.3431	-3.8178	0.4570	-0.5332	5.8311	0.0276	-2.1133	6.2777	1.9872	0.4564	3.7776	-0.9867	-0.4536	6.4507	1.1718
F	-1.6733	-2.8892	-2.3159	-2.6833	7.9269	0.1602	-4.7791	7.6762	1.4386	-1.4031	5.8848	-2.0268	0.9494	5.5284	3.7378
O	-0.8036	-1.4166	-0.0610	-1.0637	4.1128	1.7680	-1.7355	4.6731	0.1089	-0.8536	2.0403	0.2589	-1.3306	4.1988	1.7861
F	-2.1097	-4.8860	-1.5946	-3.9144	6.3845	-0.7172	-4.7758	7.7088	-0.7257	-2.3353	4.3457	-3.2270	0.4667	7.6337	3.6209
C	-2.8021	-3.0761	-0.2327	-2.8399	5.8867	1.3533	-4.1072	5.6927	0.3334	-2.1850	3.9609	-0.8874	-1.3538	6.1181	3.6571
H	-3.3711	-2.1398	-0.2820	-2.3484	6.4117	2.1749	-4.4260	5.0279	1.1455	-2.1031	4.5102	0.0548	-1.7283	5.2521	4.2144
H	-3.3641	-3.8131	0.3466	-3.5930	5.1946	1.7534	-4.2038	5.1894	-0.6326	-3.0519	3.2966	-0.8377	-1.9974	6.9799	3.8489
C	0.5048	-1.4114	-0.7203	0.3075	3.6056	1.6915	-1.3248	3.6982	1.1201	-0.1806	2.4316	1.4993	-0.0013	3.6054	1.6410
H	1.2750	-1.4824	0.0544	0.9468	4.4145	1.3255	-0.4041	4.0644	1.5853	0.3890	3.3456	1.3142	0.6012	4.2499	0.9952
H	0.5770	-2.2780	-1.3830	0.5617	3.3927	2.7328	-2.1008	3.6150	1.8860	-0.9802	2.6349	2.2181	0.4543	3.5467	2.6325
C	-2.5658	-3.6300	-1.6222	-3.5057	6.9066	0.4527	-4.9824	6.9242	0.3417	-2.3598	4.9468	-2.0233	0.0479	6.4579	4.1099
C	0.5958	-0.1083	-1.4746	0.4005	2.3656	0.8138	-1.1101	2.3860	0.3980	0.7182	1.3020	1.9590	-0.2112	2.2287	1.0499
H	1.5231	-0.1423	-2.0591	-0.0443	1.5118	1.3390	-0.9336	1.6219	1.1646	1.1694	1.6437	2.8985	0.7242	1.6739	1.1982
H	-0.2336	-0.0426	-2.1910	-0.1822	2.5215	-0.1019	-2.0375	2.1035	-0.1209	0.1100	0.4287	2.2158	-0.9871	1.7114	1.6286

Table S4. Enthalpy (ΔH) and Gibbs free energy (ΔG) of the complexation process for DIMEB:BFS complexes, obtained from theoretical calculations (BHandHLYP-D4/6-31G(d,p)) performed over the temperature range of 298.15–313.15 K.

BHandHLYP-D4/6-31G(d,p)								
**Comp.**	**298.15ΔH**	**298.15ΔG**	**303.15ΔH**	**303.15ΔG**	**308.15ΔH**	**308.15ΔG**	**313.15ΔH**	**313.15ΔG**
K1	-196.05	-99.90	-196.00	-98.30	-195.95	-96.71	-195.90	-95.12
K2	-167.62	-73.62	-167.61	-72.05	-167.59	-70.47	-167.57	-68.89
K3	-155.00	-66.60	-154.95	-65.09	-154.90	-63.58	-154.85	-62.08
K4	-163.62	-70.16	-163.56	-68.58	-163.51	-66.99	-163.45	-65.41
K5	-170.41	-83.79	-170.34	-82.29	-170.28	-80.79	-170.21	-79.30

Table S5. Enthalpy (ΔH) and Gibbs free energy (ΔG) of the complexation process for DIMEB:BFS complexes, obtained from theoretical calculations (CAM-B3LYP-D4/6-31G(d,p)) performed over the temperature range of 298.15–313.15 K.

CAM-B3LYP-D4/6-31G(d,p)								
**Comp.**	**298.15ΔH**	**298.15ΔG**	**303.15ΔH**	**303.15ΔG**	**308.15ΔH**	**308.15ΔG**	**313.15ΔH**	**313.15ΔG**
K1	-221.14	-128.12	-221.09	-126.57	-221.04	-125.02	-220.99	-123.47
K2	-184.59	-96.87	-184.54	-95.39	-184.48	-93.90	-184.43	-92.42
K3	-174.16	-86.88	-174.11	-85.38	-174.06	-83.89	-174.02	-82.40
K4	-189.95	-92.87	-189.94	-91.25	-189.92	-89.63	-189.91	-88.01
K5	-189.00	-102.26	-188.94	-100.78	-188.88	-99.29	-188.81	-97.81

Table S6. Enthalpy (ΔH) and Gibbs free energy (ΔG) of the complexation process for DIMEB:BFS complexes, obtained from theoretical calculations (PBEh-3c) performed over the temperature range of 298.15–313.15 K.

PBEh-3c								
**Comp.**	**298.15ΔH**	**298.15ΔG**	**303.15ΔH**	**303.15ΔG**	**308.15ΔH**	**308.15ΔG**	**313.15ΔH**	**313.15ΔG**
K1	-111.92	-35.01	-111.84	-33.61	-111.76	-32.22	-111.68	-30.82
K2	-86.11	-14.64	-85.99	-13.33	-85.87	-12.02	-85.76	-10.71
K3	-80.31	-2.48	-80.25	-1.08	-80.18	0.31	-80.11	1.70
K4	-78.50	-6.60	-78.38	-5.28	-78.26	-3.96	-78.15	-2.64

Table S7. Enthalpy (ΔH) and Gibbs free energy (ΔG) of the complexation process for DIMEB:BFS complexes, obtained from theoretical calculations (r^2SCAN-3c) performed over the temperature range of 298.15–313.15 K.

r^2SCAN-3c								
**Comp.**	**298.15ΔH**	**298.15ΔG**	**303.15ΔH**	**303.15ΔG**	**308.15ΔH**	**308.15ΔG**	**313.15ΔH**	**313.15ΔG**
K1	-89.71	-13.90	-89.62	-12.54	-89.53	-11.18	-89.44	-9.83
K2	-88.89	-16.12	-88.81	-14.81	-88.73	-13.50	-88.65	-12.19
K3	-77.70	-2.64	-77.64	-1.29	-77.57	0.05	-77.50	1.40
K4	-68.88	7.39	-68.80	8.75	-68.73	10.11	-68.65	11.47

Table S8. Enthalpy (ΔH) and Gibbs free energy (ΔG) of the complexation process for DIMEB:BFS complexes, obtained from theoretical calculations (r^2SCAN50-D4/6-31G(d,p)) performed over the temperature range of 298.15–313.15 K.

r^2SCAN50-D4/6-31G(d,p)								
**Comp.**	**298.15ΔH**	**298.15ΔG**	**303.15ΔH**	**303.15ΔG**	**308.15ΔH**	**308.15ΔG**	**313.15ΔH**	**313.15ΔG**

K1	-168.08	-78.51	-168.02	-76.99	-167.97	-75.48	-167.91	-73.97
K2	-140.30	-61.89	-140.23	-60.51	-140.17	-59.13	-140.10	-57.76
K3	-133.19	-49.99	-133.14	-48.55	-133.09	-47.11	-133.04	-45.67
K4	-131.90	-43.00	-131.88	-41.46	-131.86	-39.92	-131.84	-38.38
K5	-142.36	-60.99	-142.29	-59.56	-142.22	-58.13	-142.16	-56.70

Table S9. Enthalpy (ΔH) and Gibbs free energy (ΔG) of the complexation process for DIMEB:BFS complexes, obtained from theoretical calculations (ωB97X-D4/6-31G(d,p)) performed over the temperature range of 298.15–313.15 K.

ωB97X-D4/6-31G(d,p)								
**Comp.**	**298.15ΔH**	**298.15ΔG**	**303.15ΔH**	**303.15ΔG**	**308.15ΔH**	**308.15ΔG**	**313.15ΔH**	**313.15ΔG**
K1	-184.65	-94.99	-184.60	-93.48	-184.54	-91.98	-184.49	-90.47
K2	-154.91	-65.11	-154.89	-63.59	-154.87	-62.07	-154.85	-60.56
K3	-149.83	-65.18	-149.78	-63.73	-149.73	-62.28	-149.68	-60.82
K4	-152.12	-63.64	-152.07	-62.13	-152.02	-60.62	-151.97	-59.12
K5	-153.20	-70.11	-153.13	-68.68	-153.07	-67.24	-153.00	-65.81

Table S10. Enthalpy (ΔH) and Gibbs free energy (ΔG) of the complexation process for DIMEB:BFS complexes, obtained from theoretical calculations (M06-2X-D3/6-31G(d,p)) performed over the temperature range of 298.15–313.15 K.

M06-2X-D3/6-31G(d,p)								
**Comp.**	**298.15ΔH**	**298.15ΔG**	**303.15ΔH**	**303.15ΔG**	**308.15ΔH**	**308.15ΔG**	**313.15ΔH**	**313.15ΔG**
K1	-197.46	-97.23	-197.38	-95.54	-197.30	-93.87	-197.21	-92.19
K2	-175.23	-88.45	-175.15	-87.00	-175.06	-85.54	-174.97	-84.09
K3	-175.14	-79.00	-175.07	-77.38	-175.00	-75.77	-174.92	-74.17
K4	-173.14	-75.07	-173.06	-73.43	-172.98	-71.78	-172.90	-70.14
K5	-171.43	-82.79	-171.35	-81.30	-171.26	-79.81	-171.16	-78.33

Table S11. Enthalpy (ΔH) and Gibbs free energy (ΔG) of the complexation process for DIMEB:BFS complexes, obtained from theoretical calculations (M08-HX-D3/6-31G(d,p)) performed over the temperature range of 298.15–313.15 K.

M08-HX-D3/6-31G(d,p)								
**Comp.**	**298.15ΔH**	**298.15ΔG**	**303.15ΔH**	**303.15ΔG**	**308.15ΔH**	**308.15ΔG**	**313.15ΔH**	**313.15ΔG**

K1	-136.43	-88.99	-135.80	-88.20	-135.17	-87.42	-134.55	-86.65
K2	-123.23	-75.31	-122.60	-74.52	-121.98	-73.72	-121.35	-72.95
K3	-123.96	-71.60	-123.34	-70.73	-122.73	-69.86	-122.12	-69.01
K4	-113.26	-58.16	-112.65	-57.25	-112.03	-56.34	-111.41	-55.44
K5	-115.37	-70.53	-114.74	-69.78	-114.11	-69.04	-113.48	-68.32

Table S12. Enthalpy (ΔH) and Gibbs free energy (ΔG) of the complexation process for DIMEB:BFS complexes, obtained from theoretical calculations (M05-2X-D3/6-31G(d,p)) performed over the temperature range of 298.15–313.15 K.

M05-2X-D3/6-31G(d,p)								
**Comp.**	**298.15ΔH**	**298.15ΔG**	**303.15ΔH**	**303.15ΔG**	**308.15ΔH**	**308.15ΔG**	**313.15ΔH**	**313.15ΔG**
K1	-208.01	-104.32	-207.97	-102.58	-207.94	-100.84	-207.90	-99.10
K3	-175.97	-96.14	-175.89	-94.80	-175.81	-93.46	-175.72	-92.13
K4	-173.30	-85.54	-173.22	-84.07	-173.13	-82.60	-173.05	-81.13
K5	-177.24	-96.54	-177.15	-95.19	-177.05	-93.84	-176.96	-92.49

Table S13. Enthalpy (ΔH) and Gibbs free energy (ΔG) of the complexation process for DIMEB:BFS complexes, obtained from theoretical calculations (MN15-L-D3/6-31G(d,p)) performed over the temperature range of 293.15–313.15 K.

MN15-L-D3/6-31G(d,p)										
**Comp.**	**293.15H**	**293.15H**	**298.15H**	**298.15G**	**303.15H**	**303.15G**	**308.15H**	**308.15G**	**313.15H**	**313.15G**
K1	-212.68	-148.57	-212.35	-147.48	-212.01	-146.40	-211.67	-145.32	-211.33	-144.25
K3	-196.92	-131.98	-196.61	-130.87	-196.30	-129.77	-195.98	-128.68	-195.67	-127.59
K5	-185.44	-127.28	-185.11	-126.29	-184.78	-125.31	-184.44	-124.33	-184.11	-123.36

Table S14. Enthalpy (ΔH) and Gibbs free energy (ΔG) of the complexation process for DIMEB:BFS complexes, obtained from theoretical calculations (B3LYP-D4/6-31G(d,p)) performed over the temperature range of 298.15–313.15 K.

B3LYP-D4/6-31G(d,p)								
**Comp.**	**298.15ΔH**	**298.15ΔG**	**303.15ΔH**	**303.15ΔG**	**308.15ΔH**	**308.15ΔG**	**313.15ΔH**	**313.15ΔG**
K1	-206.95	-116.60	-206.90	-115.09	-206.85	-113.57	-206.80	-112.06
K2	-173.40	-87.79	-173.35	-86.33	-173.29	-84.88	-173.23	-83.43
K3	-162.99	-80.23	-162.94	-78.80	-162.89	-77.37	-162.84	-75.94
K4	-176.93	-87.54	-176.87	-86.03	-176.82	-84.52	-176.75	-83.01
K5	-176.13	-94.22	-176.06	-92.80	-175.99	-91.38	-175.92	-89.95

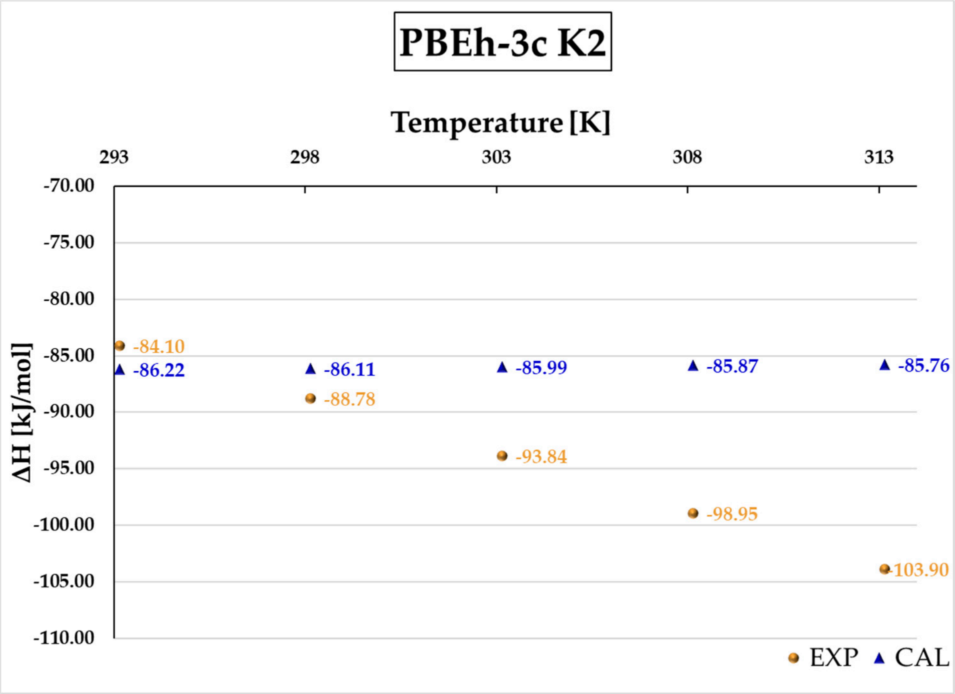

Figure S2. Enthalpy (ΔH) of the complexation process for the DIMEB:BFS complex, obtained from theoretical calculations (CAL) at the PBEh-3c level for the K2 complex and from experimental measurements (EXP) over the temperature range 293.15–313.15 K.

Table S15. The value of molar concentration of salt C_{salt} [mol/dm^3]. concentration of DIMEB C_{CD} [mol/dm^3]. molar conductivity Λ_m [S · cm^2 · mol^{-1}] for DIMEB:BFS complex in water at all tested temperatures at pressure p = 0.1 MPa.[a]

T [K]	C_{salt}	C_{CD}	293.15	298.15	303.15	308.15	313.15
Nr			Λ_m	Λ_m	Λ_m	Λ_m	Λ_m
1	0.0002869	0.000000	81.1792	95.4792	110.0826	124.3043	142.1778
2	0.0002815	0.000297	80.1878	94.4877	109.0912	123.3129	141.1864
3	0.0002763	0.000587	79.1865	93.4864	108.0899	122.3116	140.1851
4	0.0002704	0.000896	78.1324	92.4324	107.0359	121.2575	139.1311
5	0.0002620	0.001240	77.0011	91.3010	105.9045	120.1262	137.9997
6	0.0002591	0.001555	76.1328	90.4327	105.0362	119.2579	137.1314
7	0.0002535	0.001876	75.2038	89.5038	104.1073	118.3289	136.2025
8	0.0002471	0.002172	74.5625	88.8625	103.4660	117.6876	135.5612

9	0.0002421	0.002365	73.8795	88.1795	102.7829	117.0046	134.8782
10	0.0002359	0.002666	73.2353	87.5353	102.1388	116.3604	134.2340
11	0.0002321	0.002890	72.7079	87.0079	101.6113	115.8330	133.7065
12	0.0002262	0.003245	72.1726	86.4726	101.0760	115.2977	133.1712
13	0.0002205	0.003548	71.6855	85.9855	100.5890	114.8106	132.6842
14	0.0002122	0.003855	71.2817	85.5816	100.1851	114.4067	132.2803
15	0.0002071	0.004161	70.9394	85.2394	99.8429	114.0645	131.9381
16	0.0001955	0.004459	70.7004	85.0003	99.6038	113.8255	131.6990
17	0.0001931	0.004724	70.4485	84.7485	99.3519	113.5736	131.4471
18	0.0001890	0.005029	70.2780	84.5779	99.1814	113.4030	131.2766
19	0.0001832	0.005310	70.0494	84.3493	98.9528	113.1745	131.0480
20	0.0001805	0.005590	69.8780	84.1779	98.7814	113.0031	130.8766
21	0.0001739	0.005854	69.7639	84.0639	98.6673	112.8890	130.7625
22	0.0001702	0.006124	69.6047	83.9047	98.5081	112.7298	130.6033
23	0.0001850	0.006432	69.4941	83.7941	98.3975	112.6192	130.4927

[a] Standard uncertainties are $u(T)$ = 0.01 K. $u(p)$ = 0.05 MPa. $u(c)$ = 10−4·c. and the combined expanded uncertainty is Uc(Λ) = 0.0005·Λ (level of confidence = 0.95).

Table S16. The entropy (ΔS) [kJ/mol·K] of the DIMEB:BFS complex, determined from conductometric measurements over the temperature range 293.15–313.15 K.

T [K]	ΔS
293.15	-0.24
298.15	-0.26
303.15	-0.27
308.15	-0.29
313.15	-0.31
